# Powering mesoporous silica nanoparticles into bioactive nanoplatforms for antibacterial therapies: strategies and challenges

**DOI:** 10.1186/s12951-023-02093-w

**Published:** 2023-09-08

**Authors:** Biao Li, Yan Liao, Xiaoyu Su, Shuiyan Chen, Xinmin Wang, Baode Shen, Hao Song, Pengfei Yue

**Affiliations:** 1 Lab of Modern Preparation of TCM, Ministry of Education, Jiangxi University of Chinese Medicine, 1688 MEILING Avenue, Nanchang, 330004 China; 2 Australian Institute for Bioengineering and Nanotechnology, The University of Queensland, Brisbane, QLD 4072 Australia

**Keywords:** Mesoporous silica nanoparticles, Antibacterial application, Bioactive platform, Photothermal therapy, Photodynamic therapy, Targeting therapy

## Abstract

Bacterial infection has been a major threat to worldwide human health, in particular with the ever-increasing level of antimicrobial resistance. Given the complex microenvironment of bacterial infections, conventional use of antibiotics typically renders a low efficacy in infection control, thus calling for novel strategies for effective antibacterial therapies. As an excellent candidate for antibiotics delivery, mesoporous silica nanoparticles (MSNs) demonstrate unique physicochemical advantages in antibacterial therapies. Beyond the delivery capability, extensive efforts have been devoted in engineering MSNs to be bioactive to further synergize the therapeutic effect in infection control. In this review, we critically reviewed the essential properties of MSNs that benefit their antibacterial application, followed by a themed summary of strategies in manipulating MSNs into bioactive nanoplatforms for enhanced antibacterial therapies. The chemically functionalized platform, photo-synergized platform, physical antibacterial platform and targeting-directed platform are introduced in details, where the clinical translation challenges of these MSNs-based antibacterial nanoplatforms are briefly discussed afterwards. This review provides critical information of the emerging trend in turning bioinert MSNs into bioactive antibacterial agents, paving the way to inspire and translate novel MSNs-based nanotherapies in combating bacterial infection diseases.

**Graphical Abstract:**

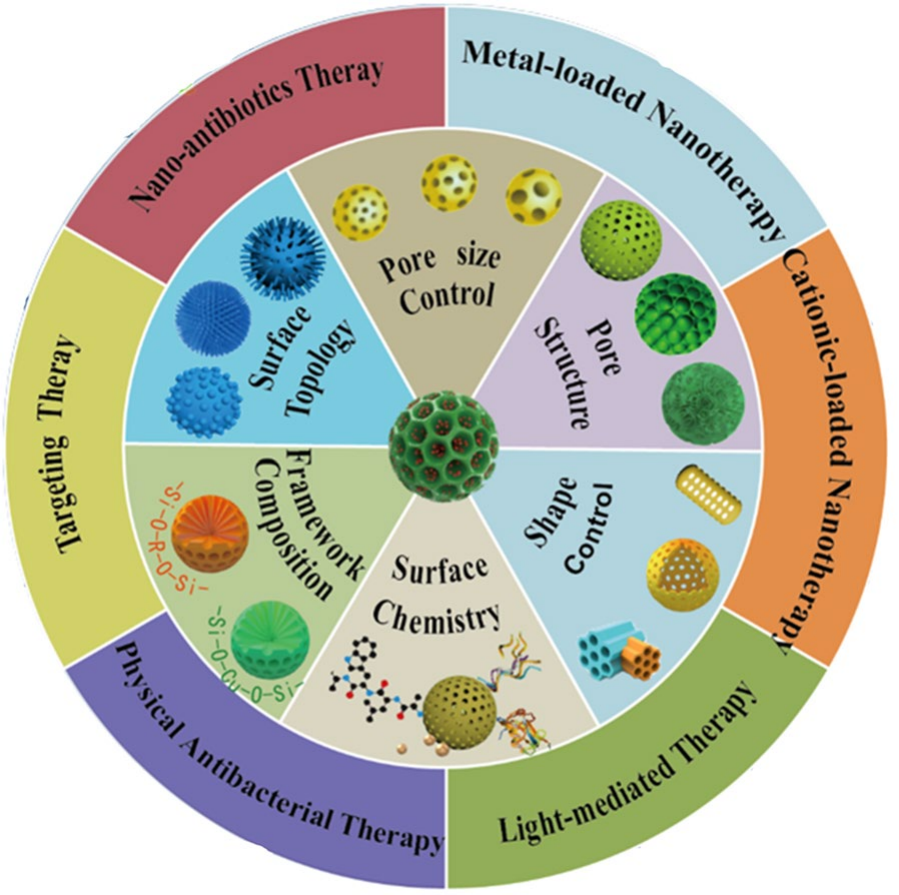

## Highlights

- Perspectives in engineering the bio-inert mesoporous silica nanoparticles (MSNs) into bio-active nanoplaltforms for antibacterial application.
- Recent research progress in the activation strategies to empower MSNs with potent antimicrobial performance.
- Outlook in the future research and translation directions in MSNs-based antibacterial therapies.

## Introduction

Bacterial infections are one of the primary illnesses that threaten human health in the world [1, 2]. Before effective control, 80% of bacterial infections are related with bacterial biofilm formation, and bacterial drug tolerance and host immune defense resistance are key causes for current antibacterial therapies [3, 4]. In general, antibiotics are the first choice in treating bacterial infections in clinics due to their outstanding bactericidal capability. However, to increase the effectiveness of antibiotics, either the dose or the frequency of administration must be increased, thus generating multidrug resistance (MDR) and side effects [5, 6]. In addition to the therapeutic challenge, bacterial infection can quickly turn into biofilms which are far more complex and harder to cure through conventional medication. Unwanted side effects and operations such as vigorous debridement of the biofilm through a physical approach would be required. To address these critical challenges, the design of novel antibacterial therapies to both improve efficacy and reduce adverse reactions holds great significance and promise in overcoming bacterial infections and antibiotic resistance.

In recent years, nanomaterials-based therapies demonstrate as potential to combat bacterial infections of difficult to treat, with the ability to avoid current pathways related to acquired drug resistance, such as metal-based nanomaterials [7], carbon-based nanomaterials [8], polymeric nanomaterials [9], and smart nanomaterials [10], etc. Among these, mesoporous silica nanoparticles (MSNs) have been considered to be one of the most potential nanocarriers for antibacterial agents, due to their unique properties, including (1) porous structures (2–50 nm) with tunable pore size and high loading capacity [11, 12]; (2) large surface area and facile surface functionalization [13, 14]; (3) gated pore can prevent premature release of the internal guest molecules [15]; (4) adjusted particles sizes and morphology, and the unique structures properties can achieve different delivery demands [16]; (5) various framework engineering strategies can enrich the biodegradation of MSNs with stimulus responsiveness [17–19]. These paramount features make mesoporous silica nanoparticles as an ideal nanoplatform shaped with multi-functionalities that can be used to treat bacterial infection, particularly achieving targeting and stimuli-responsive drug delivery [20, 21]. Zhou et al. summarized MSNs-based stimulus response design strategies, and their applications in the treatment of a wide range of diseases such as bacterial infections, cancer, diabetes, bone diseases and bone regeneration, [22]. Based on electrostatic interactions, ligand-receptor interactions, and antigen–antibody recognition, MSNs can specifically target bacteria and biofilms, and disrupt bacterial biofilm by eliminating extracellular polymeric substance (EPS) and releasing antibacterial agents under infection microenvironments, triggered by endogenous or exogenous stimulus. Therefore, enormous work has been focused on engineering the chemistry feature of MSNs to smartly transport antibacterial drugs to the location of bacterial and biofilm infection, enhance the antibacterial activity, and reduce the cytotoxicity of antibacterial agents [23, 24]. For example, Selvarajan et al. reviewed the synthesis strategy of MSNs, the antibiotic loading capacity, and their applications in targeting intracellular bacterial infections [25]. Even though, the use of MSNs for smart delivery of antibiotics and their diverse antibacterial application have been well documented, the essential structural properties and antibacterial strategies of MSNs that manipulate MSNs into bioactive nanoplatforms for enhanced antibacterial therapies have not been discussed.

**Fig. 1:**
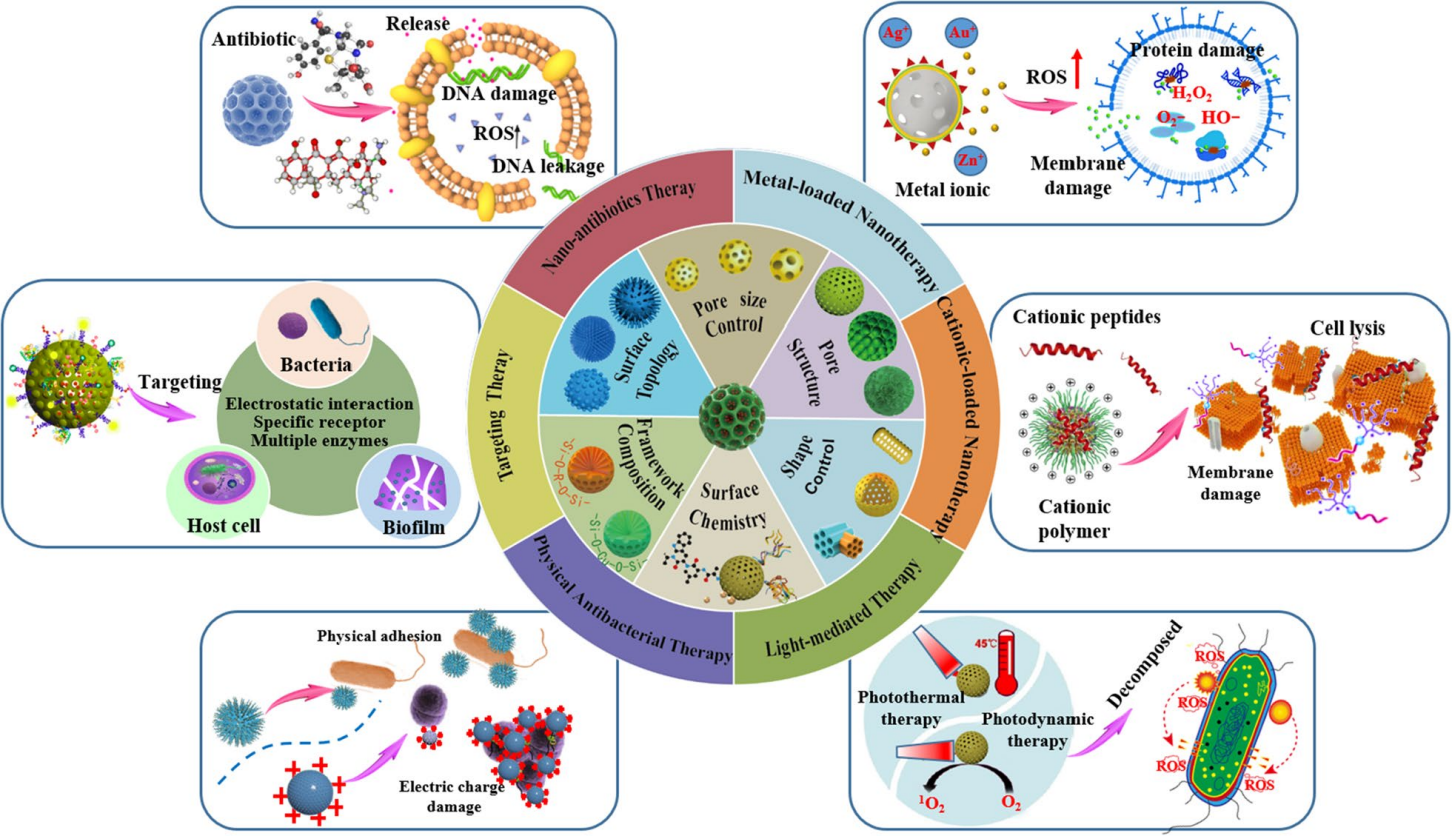
Schematic summary of the structural/chemical properties of MSNs and associated antibacterial approaches of MSNs-based nanotherapies against bacterial infection.

This review provides a unique perspective on converting the bio-inert silica into bio-active, enhancing the bacterial killing effect through various approaches beyond just delivery function. Firstly, this review critically summarizes the recent advances of these emerging research directions, with the structural properties of MSNs that are playing key roles in antibacterial processes overviewed, and novel antibacterial approaches of MSNs-based nanomaterials discussed in detail (Fig. 1). In the end, we analyze potential challenges faced by MSNs-based nanotherapies for bacterial infection treatment in the clinic, and provide our perspective on the future development directions of this field.

## Overview of the critical properties of MSNs for antibacterial therapy

The use of MSNs as nanocarriers for antibacterial therapy is a very classic strategy. It has been widely demonstrated that the diverse properties of the structure of nanomaterials can significantly impact their biomedical application [26, 27]. To this end, the critical physicochemical properties, including pore size, shape geometry, framework composition, surface chemistry, and surface topology, were discussed in detail in this section.

### Tunability of the pore size of MSNs

**Fig. 2:**
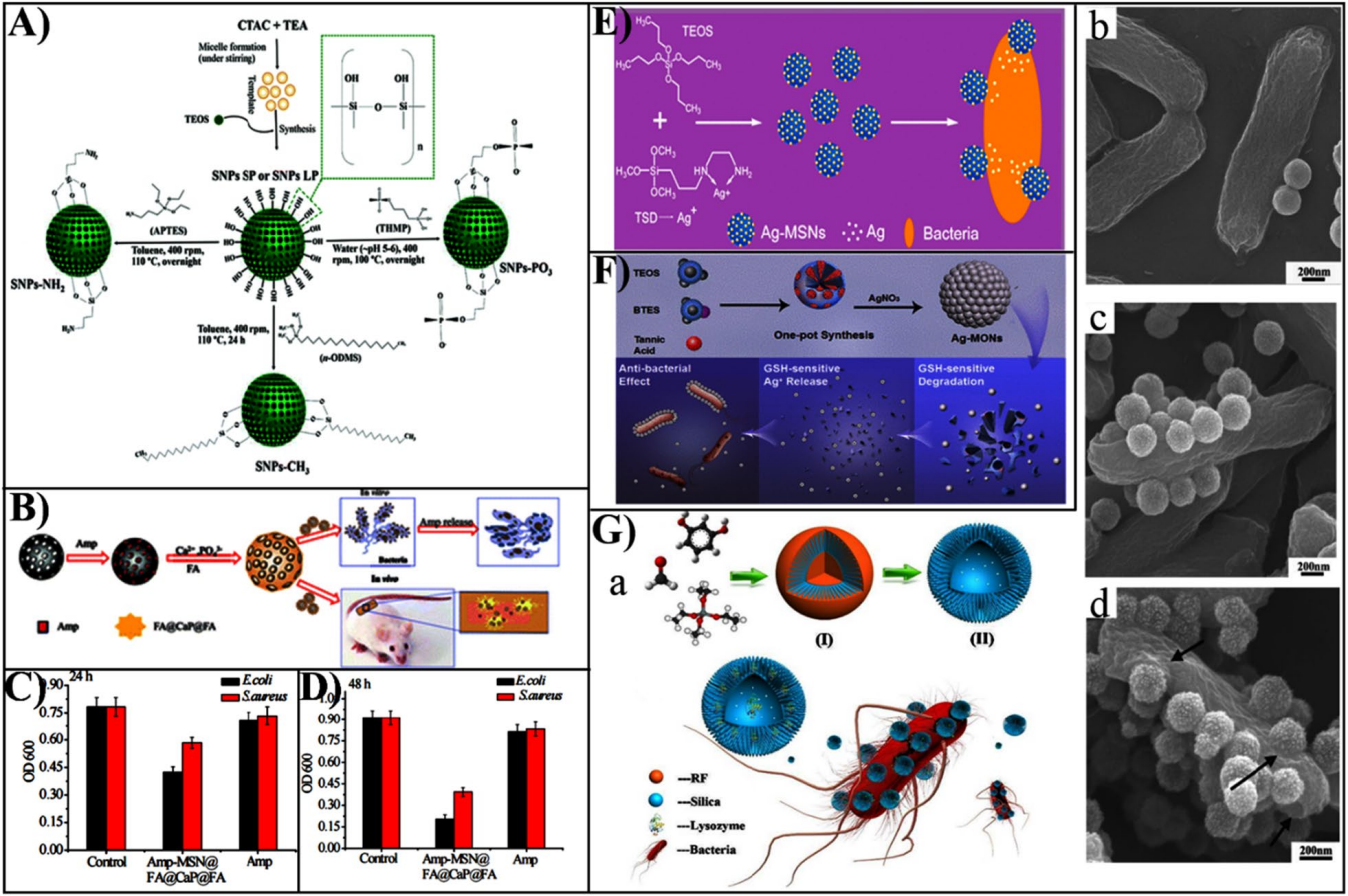
**A** Schematic synthesis of SNPs nanoparticles and the functionalization process. Reproduced with permission from Ref. [28] Copyright 2021, The Royal Society of Chemistry. **B** Schematic diagram of the design concept and evaluation of the antimicrobial properties of the composite material. **C** The composite nanoparticles and Amp interacted with *E. coli* and *S. aureus* after 24 h showing OD600 values. **D** The composite and Amp act on *E. coli* and *S. aureus* after 48 h showing OD600 values. Reproduced with permission from Ref. [43] Copyright 2018, The Royal Society of Chemistry. **E** Synthesis of silver nanoparticles decorated with mesoporous silica and their adhesion to bacteria. Reproduced with permission from Ref. [46] Copyright 2014, American Chemical Society. **F** Design of matrix degradation-based silver ion-modified mesoporous silica and its GSH response release. Reproduced with permission from Ref. [47] Copyright 2020, American Chemical Society. **G** Nanopollen-like mesoporous silica for antibacterial bacteria. **a** Schematic showing the synthesis of nanopollen-like S-SHSs and adhesion to bacteria. **b** SEM image of the synthesized S-SHSs nanoparticles. **c** Adhered on the *E. coli* surface of R-MSHSs-B, **d** R-MSHSs. Reproduced with permission from Ref. [54] Copyright 2016, American Chemical Society

The pore size and pore structure of MSNs can provide a large capacity for the loading and delivery of several antibacterial agents beyond antibiotics, such as peptide, enzymes and metal nanoparticles [21]. Ndayishimiye et al. synthesized MSNs with different pore sizes (2 nm and 9 nm), with a loading capacity of 18–29 wt% for vancomycin of a widely used natural antimicrobial peptide (Fig. 2A) [28]. Compared to MSNs with small pores, MSNs with large pores show higher load capacity and prolonged release behaviors. Peng et al. evaluated the MSNs type and pore structure on the effects of drug release properties, finding that the hexagonal pore of MSNs possessed sustained release profile [29].

Owing to the characteristics of adjustable pore size and morphology, smaller mesopores of MSN are suitable for carrying small molecules, while those with larger pore sizes are more appropriate for transporting large molecules. Wang et al. fabricated macroporous dendritic mesoporous silica nanoparticles (DMSNs) of particle sizes controllable, and the association of particle size of DMSNs with the loading and transport properties of lysozyme was also investigated [30]. The large pore size (160 nm) DMSNs or the small pore-sized (2.4 nm) MSNs showed significantly higher loading and superior antibacterial activity compared to the DMSN with a large pore size of 22.4 nm and a small pore size of 79 nm. In addition, the unique structure of DMSNs enhanced adhesion to bacterial biofilm, resulting in a higher antibacterial enzyme delivery efficiency than conventional silica with relatively smooth surfaces. Macroporous/mesoporous silica (LPMS) particles have a wide pore size and typically exhibit high drug storage capacity and fast release rates. Belbekhouche et al. simultaneously loaded nalidixic acid, chloramphenicol, and ciprofloxacin with multiple antibiotics using LPMS, and the inhibition rates of *E. coli* and *S. aureus* were 70% and 20% in vitro, respectively [31]. The conventional MSNs suffer limitations in such applications due to the low cargo-loading capacity. Hollow mesoporous silica microspheres (HMSM) provided a new type of efficient drug delivery vehicle due to the creation of a large hollow inner cavity. Poostforooshan et al. prepared an amoxicillin-loaded HMSM modified by poly (allylamine hydrochloride)/poly (anion), which showed superior drug loading capacity and release characteristics by adjusting synthesis conditions, and the inhibition rate towards *E. coli* was up to 90% within 2 h [32].

### Tunability of the shape of MSNs

The shape of the MSN has a crucial effect on interactions with bacteria as well as their behavior in vivo [33]. The morphology of MSNs could have an influence on their movement in blood circulation and on the mechanics of movement through bacterial biofilms [34]. Besides, the MSN can be designed into other particular shapes, including nanospheres [35], nanorods [36], yolk-shell MSNs [37], hollow MSNs [38], and Janus MSNs [39], in order to achieve high drug loading, efficient cellular uptake, and excellent antibacterial effect. Xu et al. have successfully synthesized a new type of rod-shaped hollow MSNs with conical pores used to delivery lysozyme to bacterial biofilms [40]. The results show that structural features with large conical pores and accessibility to the inner cavity enable the loading of up to 350 mg/g of lysozyme with a sustained release behavior. Besides, the rod-shaped geometry of lysozyme loaded hollow MSNs enhanced the removal of *E. coli* biofilm [41]. It is clear that the shape of MSNs is one of the important properties influencing their behaviors both in vitro and in vivo, and designed appropriate shape geometry will endow MSNs with the ideal antibacterial ability.

### Tunability of the surface chemistry of MSNs

It has well known that mesoporous materials can be chemical modified owing to the enriched silanol groups presented on their surface. The surface modification of MSNs is one of the most widely used strategies to diversify their biomedical functions. The silanol groups that present in MSNs surface is readily grafted by different functional groups, including specific targeting ligands and gatekeepers, to endow MSNs with increased intracellular delivery, increased accumulation at the infection site, and regulated drug release [21, 42]. Chen et al. fabricated an acid-responsive nanocarrier (MSN@FA@CaP@FA) by electrostatically attracting and biomineralizing covered folic acid (FA) as well as calcium phosphate (CaP) in MSN surface (Fig. 2B) [43]. Then, ampicillin-loaded MSN achieves specific targeting through FA that effectively increases the uptake of *E. coli* and *S. aureus* and decreases the efflux effect (Fig. 2C, D). In addition, through the design of MSN surface charge and hydrophobicity, its delivery and biological behaviors, such as release property, cell uptake, targeting ability, and antibacterial activities, can also be improved [44, 45]. The various functional modifications MSN surfaces further allowed their extensive utility for antibacterial therapy.

### Tunability of chemical framework of MSNs

Purely inorganic MSNs usually consist of a non-biologically active -O-Si-O- framework and Si–OH surface groups, which may lead to limited function. To enhance therapeutic effects, the framework of the MSNs could be designed by combining inorganic components, such as Ag, Cu, and Fe metal ions, into the framework. Tian et al. prepared mesoporous silica (Ag-MSNs) decorated with silver nanoparticles using a one-pot method (Fig. 2E) [46]. In the framework of MSNs, Ag nanoparticles between 2 and 10 nm in diameter were highly hybridized. Due to the sustainable release of silver ions, Ag-MSNs showed superior antibacterial effects against bacteria. To improve the biocompatibility of inorganic MSNs, some organic components (such as phenyl, thioether, ethylene, etc.) doped MSNs framework represents an effective strategy to adjust the inherent bioinert characteristics of MSNs. Zhang et al. used the natural polyphenol tannic acid (TA) as a non-surfactant template to fabricate biodegradable mesoporous organosilica nanoparticles (MONs) decorated with silver nanoparticles (Fig. 2F) [47]. These well-dispersed silver nanoporous silica nanoparticles (Ag-MONs) release silver ions by matrix degradation in the presence of glutathione (GSH), leading to a superior antibacterial effect on *E. coli* and *S. aureus* than non-degradable Ag-MSN, Ag NPs, and silver nitrate. For this reason, the design of the framework composition offers more possibilities to diversify the functionality of MSNs and to improve MSNs-based antimicrobial therapy.

### Tunability of surface topology of MSNs

The design of the surface topology of MSNs and its impact on biological behavior has currently of increasing interest [16, 48]. The engineered surface topology of MSNs includes virus- [49], hemisphere- [50], tree branch-like [51], and flower-like subunit topographies. This could not only improve the interaction between MSNs and cargo molecules, but also improve the interaction between MSNs and bacteria, and enhances the contact-killing ability due to its shape, which increases local adhesion and thus leads to a change in membrane surface tension and membrane damage [51, 52]. Ahmad Nor et al. fabricated the hollow MSNs with regulated surface roughness. The results showed that the rough MSNs which have hydrophilic composition exhibited suprisingly hydrophobicity, leading to superior loading of hydrophobic vancomycin and enhanced antibacterial effect, compared to the smooth surface MSNs [53]. Inspired by the adhesion of pollen grains to hirsute insects, we designed a nano-pollen-like MSN with a spike-like surface, which has enhanced adhesion to bacteria compared to the smooth surface MSN (Fig. 2G) [54]. Furthermore, nano-pollen-like silica loaded with lysozyme was found to have stronger antibacterial activity against *E. coli*, due to high local enzyme concentrations enabled by the enhanced adhesion (Fig. 2G). Thus, due to the enhanced adhesion to the bacterial surface caused by the rough surface, the MSN surface topography has been broadly considered to be a critical feature affecting surface-bacteria interactions.

## Functionalization of MSNs for enhanced antibacterial therapies

MSNs represent a suitable choice for the development of antibacterial biomaterials due to the above-mentioned distinctive features, and the most straightforward approach is to utilize the porous structures of MSNs to deliver antibiotics for infection control [55]. However, bare MSNs show simply a hydrophilic surface property with rich hydroxyl groups, which would fit a small range of antibiotics to adsorb on the surface. Considering the diverse physicochemical features of a library of antimicrobial agents, functionalization of MSNs to endow themselves with tailorable surface properties become an effective strategy to match the cargo molecules [56]. By introducing different functional groups on the surface or framework of MSNs, the interaction between MSNs and antibacterial agents or bacteria can be enhanced, drug release can be regulated, therefore the biological activity of MSNs-based antibacterial therapy can be improved. To this end, a variety of functional groups have been modified on MSNs to fit the properties of antimicrobials such as conventional antibiotics [57], metal compounds [58], cationic polymers [59], and antibacterial peptides (Table 1) [60]. Additionally, due to unique physicochemical properties, MSNs-based physical antibacterial therapy can also interact with bacterial cell membranes through specific interactions, resulting in the damage of bacterial membranes and causing leakage of cytoplasm.

**Table 1:** Antibacterial agents-loaded MSN nanoplatforms for antibacterial application

Antibacterial strategies	Nanocarriers	Tested microorganism	Therapeutic effects	Refs.
Antibiotic delivery	MSNConA@LEVO	*E. coli*	Effective penetration in Gram-negative bacterial biofilm	[66]
	PMB^B/N/C-MSN	*E. coli*	High loading capacity, enhance antibacterial activity	[68]
	MSN-NH_2_-CEF/MEP	*A. baumannii*	Enhance bactericidal activity, reduce side effects and toxicity	[69]
Silver-incorporated	CCM@SBA-15/PDA/Ag	*E. coli*,* S. aureus*	Reduce side effects, enhance Gram-negative bacterial killing	[72]
	MSN-Ag-DNase I	*E. coli*,* S. mutans*	Enhance bacterial biofilm eradication	[73]
Copper-incorporated	SBA-CuO	*E. coli*	Stronger antibacterial properties	[76]
	MSN-maleamic-Cu	*E. coli S. aureus*	Exhibiting a powerful antibacterial effect	[78]
	AZOX@MSNs-PDA-Cu	*Pyricularia oryzae*	Provides a new way to design antibacterial agents	[79]
Zinc-incorporated	ZnO-SBA-15	*E. coli*	Coordination effect assist for anti-bacterial application	[85]
	ZnO-SiO_2_	*Candida albicans*, *S. aureus*	Reduce nanoparticle retention risk in host and environment	[86]
Cationic polymer-grated	Spiky silica nanocomposite I	*Staphylacoccus Epidermidis*	Enhanced anti-microbial and anti-biofilm properties	[88]
Antibacterial peptide-grafted	MSNs@OVTp12@Gen	*E. coli*	Treatment of bacterial infections effectively and prevention of bacterial drug resistance	[93]
	MSN@T7E21R@HD5@SCN	*E. coli*	Promising oral antibacterial formulation against *E. coli*	[95]

### Tailoring the physicochemical features of MSNs for antibiotics delivery

The primary mechanisms of antibacterial activity of antibiotic-loaded MSNs were dependent on antibiotics themselves, including damaging cell membranes, causing leakage of bacterial contents, and inhibiting the synthesis of genetic material [61]. During the last decade, a large variety of antibiotics emerged, while their antimicrobial activity is basically concentration-dependent and time-dependent [62]. Particularly considering many of the antibiotics have a short half-life, while chronic infection diseases require maintaining a high antibiotic level at the infection site for a relatively longer time, controlled antibiotic release through MSNs presents an ideal solution to this issue. A number of antibiotics with potent antibacterial activity but quick clearance rate, such as tetracycline (TC), vancomycin (VAN), azithromycin (AZ), etc. have been used to load into MSNs for sustained release to manage effective bacterial inhibition and eradication. It is well known that the fluoroquinolone drug ciprofloxacin (CIP) is preferred for the treatment of *Salmonella* infections, but high-dose and repeated use leads to reduced susceptibility and numerous adverse effects. Alandiyjany et al. evaluated the efficacy of mesoporous silica loaded with ciprofloxacin in rats infected with *Salmonella * in vivo. The mesoporous silica could regulate the release of CIP to prolong the antibacterial effect, and the clearance of *Salmonella typhi* biofilm was over 50% [63].

Among the different organic antibacterial agents, such as quaternary ammonium salts, peptides, guanidine, and phosphonium salts, have been widely used to enhance antibacterial ability. Among these, non-halogenated amines have been widely applied in antibacterial materials. The grafting of the *N-*haloamine precursor monomer 3-(3′-hydroxypropyl)-5,5-dimethylhydantoin (APDMH) to the surface of the mesoporous material allows for a powerful antibacterial effect [64]. The antibacterial mechanism of *N-*halamine was the transfer of mesoporous silica nanoparticles to the bacterial cell wall through halogen atoms, thereby disrupting intracellular receptors and inhibiting enzymatic or metabolic processes in order to inactivate the bacteria. Carbazole derivatives also have been reported to have strong antibacterial activity, Ankita et al. loaded carbazole monomers with styrene and vinyl-modified mesoporous silica to test the inhibition performance against *S. aureus*, *Staphylococcus pyogenes*, *E. coli*, and *Staphylococcus typhi* [65]. It was discovered that the mechanism of the antibacterial effect of the nanoparticles might be associated with the destruction of bacterial cell membranes.

The formation of biofilms by bacteria is the main obstacle to the treatment of antibiotics owing to the difficulty of penetration into the bacterial biofilm to kill bacteria. To promote the delivery of antibiotics into the bacterial biofilms, Martínez-Carmona et al. designed a novel targeted antibacterial nanoplatform of mesoporous silica loaded with levofloxacin (LEVO) modified by the lectin Concanavalin A (ConA) [66]. This nanoplatform increased the antibacterial efficacy of the antibiotic hosted within the mesopores and reduced side effects. Currently, a combination of antibiotics is used to treat persistent or multi-bacterial infections that cannot respond to conventional treatment plans. Combination of multiple antibiotics together might broaden the range of their antibacterial activity as well as have a synergistic antibacterial impact [67]. Polymyxin B is a lipopeptide antibiotic produced by *Mycobacterium polymyxa*, but the clinical application is limited by nephrotoxicity and neurotoxicity.

**Fig. 3:**
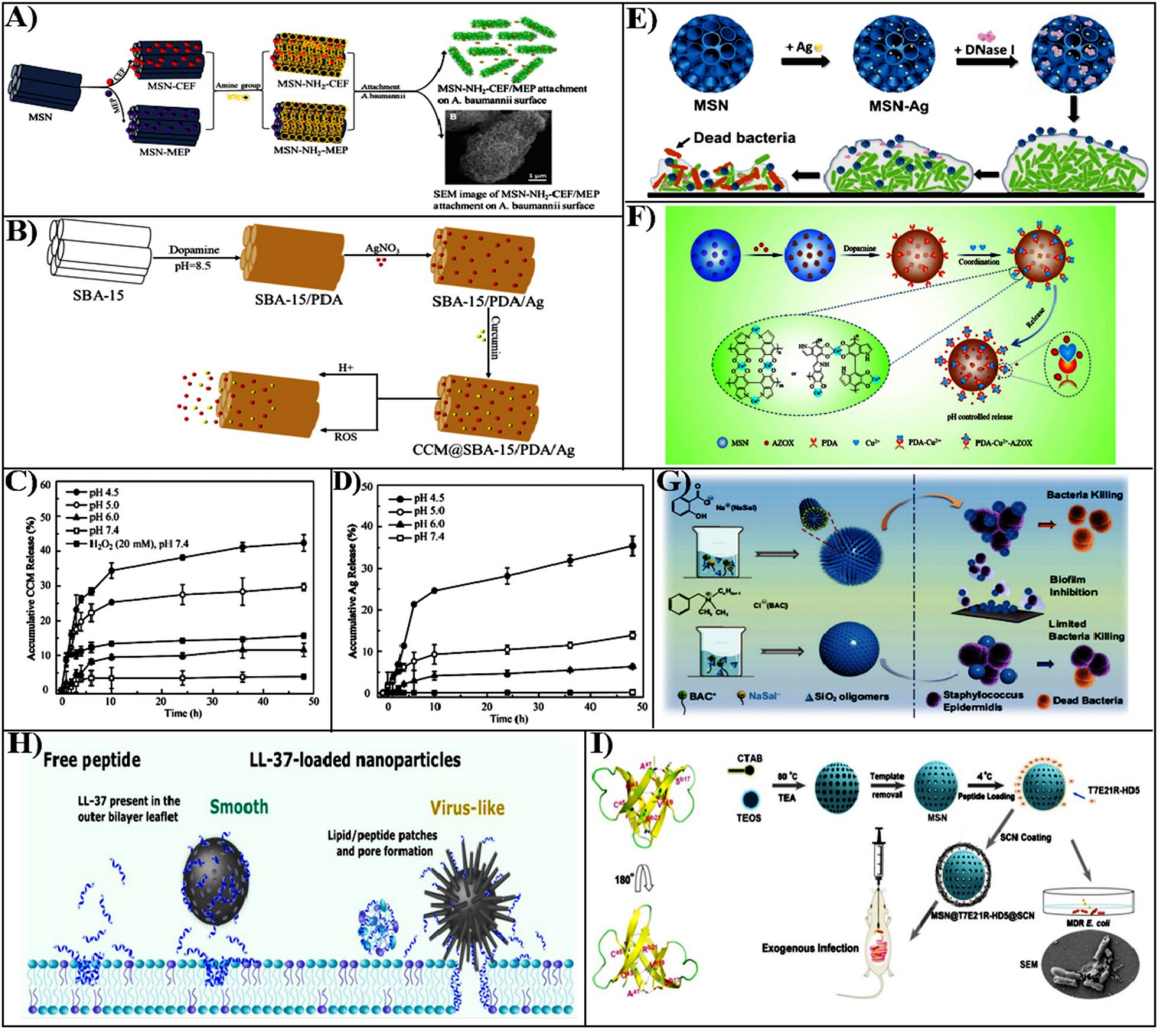
**A** Cefepime or meropenem loaded amine-functionalized mesoporous silica nanoparticles (MSN-NH_2_-CEF/MEP) against *Acinetobacter baumannii.* Reproduced with permission from Ref. [69] Copyright 2021, Elsevier B.V. **B** The design of CCM@SBA-15/PDA/Ag nanoparticles and their responsive drug release evaluation. **C** The evaluation of CCM pH, ROS response release. **D** To evaluate pH-responsive Ag release from nanoformulations. Reproduced with permission from Ref. [72] Copyright 2020, American Chemical Society. **E** The construction of Ag- and DNase I-loaded mesoporous silica and the evaluation of antibacterial and removing biofilms. Reproduced with permission from Ref. [73] Copyright 2020, RSC Pub. **F** The MSN design and release mechanism of dopamine chelated copper ions. Reproduced with permission from Ref. [79] Copyright 2020, Elsevier B.V.** G** The antibacterial and anti-biofilm activities of spiky nanocomposites synthesized by BAC and NaSal co-templates. Reproduced with permission from Ref. [88] Copyright 2022, Shanghai Jiao Tong Univ Press. **H** Illustration of the viral type and smooth MSN loaded with LL-37, and free LL-37 interaction with the cell membrane. Reproduced with permission from Ref. [94] Copyright 2021, American Chemical Society. **I** The T7E21R-HD5 structure and the design process of MSN@T7E21R@HD5@SCN. Reproduced with permission from Ref. [95] Copyright 2019, The Royal Society of Chemistry

To reduce the cytotoxicity and improve the antibacterial effect, carboxy decorated MSN loading with polymyxin B and vancomycin was fabricated, which was efficient for bacteria [68]. This nanoparticle loaded with antibiotic molecules has enhanced effectiveness of antibacterial activity in different Gram-negative bacteria compared to free antibiotics, and can reduce cytotoxicity by reducing the production of reactive oxygen species (ROS). The widespread utilization of antibiotics is promoting the design and development of antibiotic as well as nanobacterial formulations. *Acinetobacter baumannii* is resistant to several antibiotics, an effective antibiotic against this bacterial infection is a combination of polymyxin and tigecycline, Najaf et al. developed cefepime (CEF) and meropenem (MEP) loaded amine-functionalized mesoporous silica nanoparticles (MSN-NH_2_) to enhance combating bacterial infection (Fig. 3A) [69]. The MSN-NH_2_ loaded CEF and MEP exhibited a stronger antibacterial activity compared with free drugs.

### Metal-incorporated MSNs for antibacterial therapy

Owing to the unique properties such as a broad antibacterial spectrum, length of effective antibacterial period, low toxicity, absence of drug resistance, and excellent safety, the metal ions can be utilized as an effective antibacterial agent. The commonly used antimicrobial metal ions are silver ions, copper ions, zinc ions, divalent iron ions and aluminum ions. Their mode of application is typically through the leaching from their metal crystal or metal oxide nanoparticles. Metal compounds can also be incorporated into the surface of MSNs that allow a controlled release of metal ions to achieve their antimicrobial functions. The other type of metal-based antimicrobial materials is photocatalytic nanomaterial, such as nanoparticles of zinc oxide and titanium dioxide, which rely on light excitation to induce strong oxidative radicals for bactericidal properties. The metal elements can be adsorbed on the bacterial surface when they are dissolved from the carrier material in an ionic state, which can trigger bacterial death by chelating with DNA and proteins within bacterial cells, or through oxidative stress, ultimately to bacterial death [70]. Therefore, the immobilization of metal ions on the surface or inside the MSNs by physical adsorption or ion exchange, which in turn improves the bactericidal properties and stability of the metal ions. Furthermore, synthesizing cationic polymers modified MSN to effectively adhere negatively charged bacterial biofilm is also an effective antibacterial strategy.

#### Silver-incorporated MSNs-based antibacterial therapy

The silver nanoparticles are considered potential antibacterial agents for low toxicity, excellent cost-effectiveness, and wide-spectrum effectiveness [71]. Unfortunately, the application is limited by the tendency of silver nanoparticles to easily aggregate. Biomimetic polymers including polydopamine (PDA), algae, and tea polyphenols are abundant in chemically active groups, which can provide an excellent reduction platform for silver nanoparticles. Based on this strategy, Song et al. designed mesoporous silica loading silver nanoparticles and encapsulated by melanin-like polydopamine (PDA) as nanocarriers (SBA-15/PDA/Ag) (Fig. 3B) [72]. Additionally, curcumin (CCM) was loaded into SBA-15 with PDA coating (CCM@SBA-15/PDA/Ag) by non-covalent interaction, an integrated dual-responsive nanoplatforms was used for combating infectious bacteria (Fig. 3C, D). The CCM@SBA-15/PDA/Ag composite nanoparticle could enhance against Gram-negative bacteria, which could be owing to the enhanced effect of Ag interaction with the bacterial cell membrane.

The pathogenic bacterial biofilms are composed of microbial cells that accumulate on surfaces, which are thought to be a major cause of chronic bacterial infections. To improve this problem, Tasia et al. provided a novel approach to enhance the efficacy of eradication bacterial biofilms, which used large mesoporous silica doped with Ag nanoparticles and loaded with DNase I (MSN-Ag-DNase) (Fig. 3E) [73]. Compared to silver nitrate, which only reduced 53.7% of *E. coli*, MSNs-Ag-DNase I killed 73.1% of *E. coli* at a concentration of 200 g/mL and enhanced biofilm eradication. The nanoparticles significantly improved the antibacterial activity through reduced Ag nanoparticle aggregation, eliminating the extracellular DNA of EPS in collaboration with deoxyribonuclease I, and breaking the biofilm matrix enzymes.

#### Copper-incorporated MSNs-based antibacterial therapy

The antibacterial properties of copper-based antibacterial materials are similar to those of silver, but due to low cost and superior antibacterial capabilities, research on copper-based antibacterial materials has advanced significantly in recent years [74]. The antibacterial mechanism of copper ions is to make direct contact with the bacterial outer membrane to rupture, thus allowing unimpeded access of copper ion flow to the cell interior and inhibiting bacteria by affecting intracellular enzymes and protein metabolism. The primary categories of copper-based antibacterial nanomaterials are copper-loaded antibacterial agents, copper-monolithic antibacterial agents, oxide-based antibacterial agents, and copper-compatible material antibacterial agents [75]. Here, we focus primarily on introducing antibacterial agents based on MSNs loading or adsorption of copper ions.

The metal nanoparticles are contaminating the soil microbial communities, thus limiting metal ions' migration to the environment is crucial, Laskowski et al. proposed a new class of antibacterial materials by immobilizing copper ions in SBA-15 pores through propylphosphonate units [76]. Owing to the immobilization of functional groups the compounds are safer for the environment than commonly used antibacterial agents. In specific, antibacterial activity of cooper-doped SBA-15 remained to be high even with a low content of copper. Compared to only copper ions, the SBA-15-Cu containing 5% copper ions had stronger antibacterial activity against *E. coli*. Maleic acid inhibits the biological function of maleic acid amide hydrolase in bacteria, reducing bacterial viability [77]. To explore its potential application as an antibacterial agent, Díaz-García et al. successfully synthesized a MSN containing a maleimide ligand capable of coordinating with copper(II) ion [78]. The antibacterial activity of *S. aureus* and *E. coli* was significantly enhanced after coordination with copper(II) ions compared to MSN-maleic acid. This nanoplatform was able to trigger the production of large levels of ROS during oxidative stress in *E. coli* and *S. aureus*, exhibiting a powerful antibacterial effect.

Metal ion release behavior is extremely connected to carrier material and formulation technology, Xu et al. reported that copper ions (Cu^2+^) were chelated to the MSN via dopamine for the control of azoxystrobin (AZOX) release (Fig. 3F) [79]. Among these, the polydopamine coating on the MSNs surface can block the pore channels and promote the binding of copper ions. Additionally, the introduction of copper ions would restrict the release of AZOX into the environment owing to the coordination bonding interaction of copper ions and AZOX. On the other hand, copper chelation could confer a pH- responsive release patterns since H^+^ to PDA or OH to Cu^2+^ compete for coordination, breaking the PDA-Cu^2+^-AZOX coordination architecture. This strategy provides a new way to design pH-responsive systems and antibacterial pesticide applications.

#### Zinc-incorporated MSNs-based antibacterial therapy

In recent years, zinc has been widely used as an antibacterial agent owing to its unique antibacterial mechanism, including that adsorbing in bacterial cell walls, interfering with cell membranes, and causing bacterial death [80, 81]. Additionally, zinc also can trigger the generation of ROS, such as hydrogen peroxide (H_2_O_2_), hydroxyl radicals (·OH), oxygen anions, and hydro-peroxides [82, 83]. It is well known that zinc oxide is most effective against bacteria when the pH was 7–8 [84]. However, pure nano- ZnO is prone to aggregate during sterilization, which reduces the contact surface area and ROS generation, and affects the bactericidal action [82]. The loading of ZnO into the SBA-15 can effectively improve its dispersibility. The water-soluble polyethyleneimine (PEI, MW = 600 Da) modified SBA-15 could form strong coordination bonds with Zn^2+^, and adsorb zinc ions into the mesoporous silica [85]. The combination of the imine group contributed to the adsorption of more Zn^2+^ to improve dispersibility. Therefore, the PEI-coated SBA-15 loaded zinc ions were of great reference to develop antibacterial ZnO nanoparticles. In order to promote nucleation and growth of ZnO nanoparticles as well as to prevent agglomeration, Donnadio et al. prepared ZnO-SiO_2_ composites using Cab-O-Sil-H5 and Syloid 244 FP silica as carriers and evaluated their antibacterial activity [86]. This strategy can effectively reduce the dispersion risk of nanoparticles in the host and environment, which is of great value in the preparation of nanomaterials with antibacterial and antifungal activities.

### Cationic polymer functionalized MSNs for antibacterial therapy

Due to bacteria generally having negatively charged cell membranes composed of lipid layers and peptidoglycan, engineering a positive surface charged of originally negatively-charged MSNs could also enhance the interaction with the bacterial surface [87]. It was known that the antibacterial activity of cationic surfactants or polymers micelles was due to the electrostatic interaction of the positive charge head group and negatively charged bacterial membrane, as well as the lipophilic tail group which enhanced membrane permeability. Under this principle, the surface modification of MSNs, such as synthesis with cationic surfactants as templating agents or modification with cationic polymers, can provide a positive charge on the MSN surface to enhance targeting and internalization of bacteria. Benzalkonium chloride (BAC) is a bactericidal agent that has recently been used as a cationic surfactant and a template agent for the synthesis of MSN composite material. We have recently prepared a MSN composite with a spiky surface by employing a cationic structure guide (BAC) and an anionic structure guide sodium salicylate (NaSal) as a dual template to enhance adhesion and physical damage to bacterial biofilm (Fig. 3G) [88]. The spiky MSNs we synthesized with BAC and NaSal as co-templates showed higher BAC loading, and 70% biofilm inhibition against Gram-positive bacteria *Staphylococcus epidermidis* with loss of bacterial cell membranes, compared to MSNs with a smooth surface synthesized with pure BAC template. The rough MSNs with the "dual active" templates showed superior bactericidal ability, probably attributed to the disruption of the bacteria membrane through enhanced adhesion on the spiky surface that promoted the release of the dual antibacterial agents (BAC and NaSal). This MSN offers  a novel approach to manufacture of new functional MSN composites with significant antibacterial properties because they can simultaneously release two bactericidal components and generate synergistic effects on bacterial death and biofilm eradication.

### Antibacterial peptide grafted MSNs for antibacterial therapy

Antimicrobial peptides (AMPs) are a class of amphiphilic, positively charged natural peptide compounds isolated from animals, plants, and microorganisms [89, 90]. In recent years, AMPs have been considered an alternative to traditional antibiotics owing to their ability to target bacterial membranes effectively inhibit multidrug-resistant (MDR) bacteria [91]. Unlike antibiotics, AMPs mainly combine with negatively charged lipopolysaccharide layers on the cell membranes through electrostatic and hydrophobic interactions and then induce membrane rupture, which is thought to be the primary antibacterial mechanism of AMPs [92]. The AMPs not only have a significant antibacterial effect but also do not interact with bacteria surface-specific receptors. As a result, there is very rarely drug resistance to this strategy observed with treatments. A new antimicrobial peptide (OVTp12) was obtained from egg white ovotransferrin, which not only exhibited promising antimicrobial activity but also altered bacterial cell membrane permeability and morphology.

Besides, OVTp12 has high specificity for bacterial cell membranes and can be used as a target ligand for tracking bacteria, Ma et al. used OVTp12 modified MSNs loaded with gentamicin (MSNs@OVTp12@Gen) to promote the targeted bacterial cells. Compared to free Gen, the MSNs@OVTp12 nanoparticles enhance the interaction with *E. coli* cell membranes and can also effectively treat bacterial infections in vivo, significantly reducing the inflammatory response [93]. Despite the advantages of AMPs for antibacterial applications, they are weakly selective for strains and potentially toxic to the host. MSN can be used as effectively delivery vehicle for AMPs owing to its large cavities and controllable pore structure. Haffner et al. used smooth surface and virus-like MSN with ‘‘spiky’ characteristics as carriers to load the antimicrobial peptide LL-37, respectively (Fig. 3H) [94]. Through comparing the effect of two different types of mesoporous silica particles on bacterial membranes, which was found that virus-like MSN loaded with LL-37 were more disruptive on bacterial membrane and also higher than free LL-37. Furthermore, the AMPs are composed of amino acids, which are susceptible to proteases and physiological environmental conditions, leading to a significant reduction in antibacterial activity in vivo. To reduce the loss of amp in the stomach, Zhao et al. modified MSN with a succinylated casein (SCN) that can be specifically degraded by intestinal proteases for loading potent bactericide designed by site mutations at enteric (HD5 T7E21R-HD5) (Fig. 3I) [95]. The SCN coating reduces the release of T7E21R-HD5 from MSN in an acidic environment. This study provides MSN-based oral delivery strategy for AMPs to combat intestinal infection.

## Photo-synergized MSNs-based antibacterial therapies

### Photothermal therapy assisted with MSNs-based antibacterial

Photothermal therapy (PTT) is an effective approach for treatment of bacterial infections, which depends on the photothermal agents (PTAs) rapid generation of large amounts of heat when exposed to light irradiation [96, 97]. The antibacterial therapy of PTT mainly relies on photothermal agents (PTAs) that adhere to the surface of bacteria through multiple interactions and generate local heat on the surface of bacteria induced by light, which denatures the proteins on the surface of bacteria, subsequently terminates a large number of intracellular reactions, ultimately leading to bacterial death [98, 99]. Among, PTT gaining more attention in antibacterial design and biofilm eradication based on near-infrared radiation (NIR), which converts NIR light energy to heat and raises the bacterial solution temperature, inactivating the enzymes on the surface of bacteria [100–102].

**Fig. 4:**
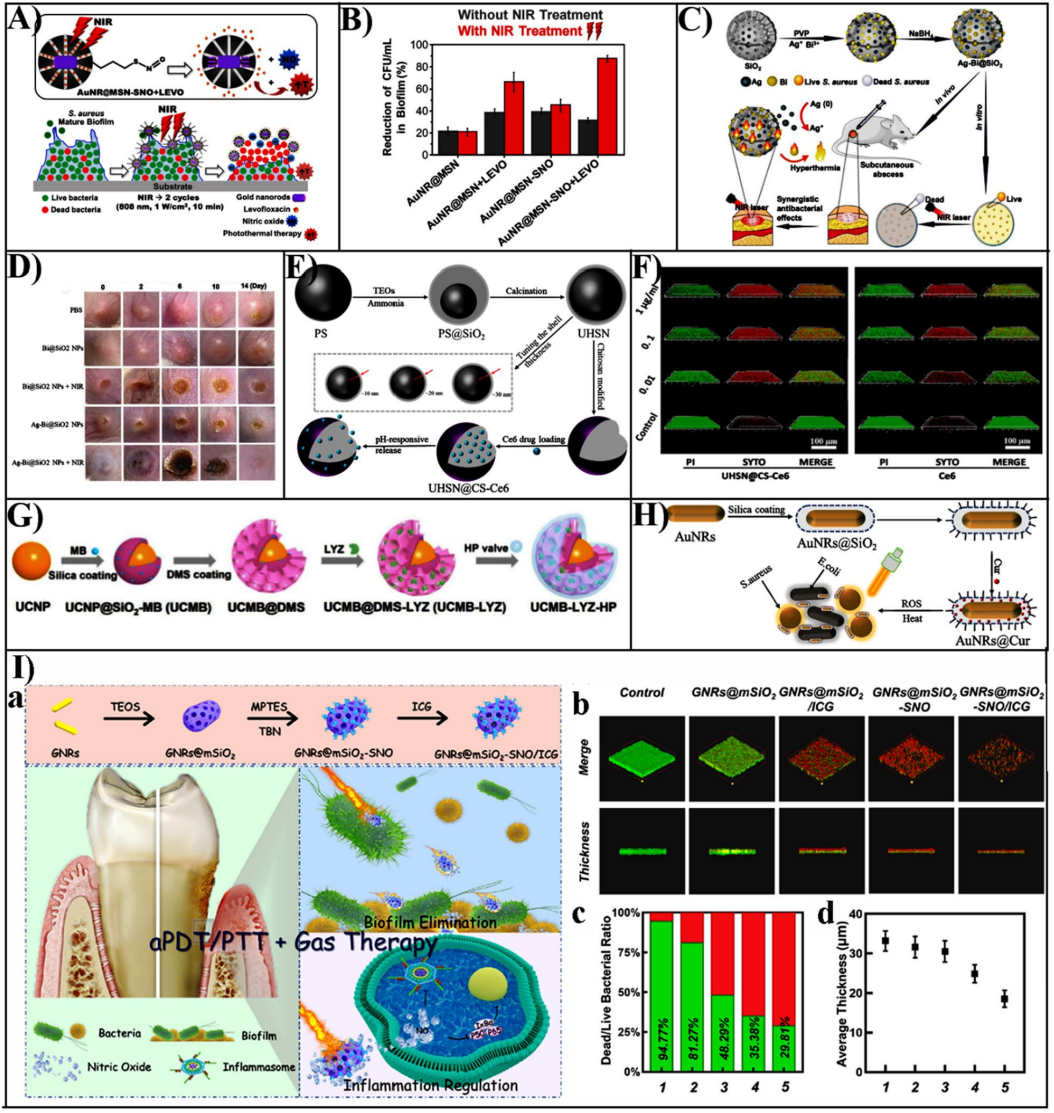
**A** Illustration of the design of NIR-responsive nanosystems and the removal of the *S. aureus* biofilms. **B** The effect was of different AuNR@MSN nanosystems photothermal on the cell viability of *S. aureus* biofilms. Reproduced with permission from Ref. [103] Copyright 2021, Elsevier Inc. **C** Schematic representation showing the preparation of Ag-Bi@SiO_2_ NPs nanoparticles and the synergistic antibacterial effect both in vitro and in vivo. **D** The healing effect of Ag-Bi@SiO_2_ NPs nanoparticles on skin wounds of *S. aureus*-infected mice. Reproduced with permission from Ref. [104] Copyright 2020, WILEY–VCH. **E** Illustration of the preparation of ultrathin hollow silica nanoparticles modified with chitosan for photodynamic antibacterial loaded with the photosensitizer Chlorin e6 (Ce6). **F** The inhibition of *S. aureus* by Ce6 and UHSN@CS-Ce6 and the elimination of biofilm. Reproduced with permission from Ref. [112] Copyright 2021, Elsevier B.V. **G** Illustration of the design of a hybrid nanosystem with photodynamic synergy. Reproduced with permission from Ref. [114] Copyright 2021, Wiley–VCH. **H** The construction of AuNRs@Cur nanocomplexes with combined photothermal and photodynamic antibacterial activity. Reproduced with permission from Ref. [123] Copyright 2021, Elsevier B.V. **I** The design of GNRs@mSiO_2_-SNO/ICG NPs nanoparticles and the role of removing bacterial biofilms. a Schematic illustration of the design of GNRs@mSiO_2_- SNO/ICG NPs and their targeted removal of periodontal bacterial biofilm as well as inflammatory modulation. b Removal of *P. gingivalis* biofilm by GNRs@mSiO_2_, GNRs@ mSiO_2_/ICG, GNRs@mSiO_2_-SNO, and GNRs@mSiO_2_-SNO/ICG nanoparticles. c Ratio of dead/live bacteria in *P. gingivalis* biofilm by GNRs@mSiO_2_, GNRs@mSiO_2_/ICG, GNRs@ mSiO_2_-SNO, GNRs@mSiO_2_-SNO/ICG nanoparticles. d The average thickness of GNRs@ mSiO_2_, GNRs@mSiO_2_/ICG, GNRs@mSiO_2_-SNO, and GNRs@mSiO_2_-SNO/ICG after the action of *P. gingivalis* biofilm. Reproduced with permission from Ref. [128] Copyright 2021, Elsevier B.V

Although PTT shows promising prospects in antibacterial approaches, excessive temperature or delocalized heat usually leads to severe damage to healthy tissues. Combination of PTT and other antibacterial agents seems to be beneficial strategy to enhance efficiency and lower side effects. García et al. developed a multifunctional hybrid organic–inorganic MSNs for loading antibiotic levofloxacin (LEVO), while nitric oxide was integrated and modified onto the MSN through nitroso (-SNO) to construct a NIR stimulus-responsive release system. (Fig. 4A) [103].

This nanoplatform removes only 30% of the biofilm of *S. aureus* biofilm when irradiated without light, but a 90% reduction when irradiated with near-infrared (NIR) (Fig. 4B). These results suggest that the development of near infrared light stimulation in response to photothermal therapy and the combination of the antibiotic levofloxacin and nitric oxide to disrupt the integrity of bacterial biofilms could lead to powerful antimicrobial therapies.

Besides, Cao et al. described a silver-bismuth (Ag-Bi@SiO_2_) NPs antibacterial agent that was supported by MSN (Fig. 4C, D) [104]. When exposed to near-infrared laser irradiation, the thermal energy generated by the Bi NPs could dissolve bacterial biofilms and promote Ag ions release, thereby enhancing antibacterial efficacy. Moreover, under NIR irradiation, the 100 µg/mL Ag-Bi@SiO_2_ NPs were effective in eliminating 69.5% of mature MRSA biofilms, which was stronger than the untreated Bi@SiO_2_NPs (26.8%) and Ag-Bi@SiO_2_NPs (30.8%). Therefore, the nanoparticles possessing photothermal antibacterial activity were a promising antibacterial platform to treat bacterial infections. Owing to their outstanding photothermal features, gold-silver nanocages (Au–Ag NCs) are widely used for PTT treatment. Wu et al. designed a gold-silver nanocage (Au–Ag@SiO_2_ NCs) nanoparticle encapsulated by silica to achieve controlled release of silver ions and enhanced bactericidal properties under NIR laser irradiation [105]. Under NIR irradiation, the nanoparticles exhibited minimum bactericidal concentration (MBC) values of 256 and 512 g/mL against *S. aureus* and *E. coli*, respectively. In contrast, Au–Ag@SiO_2_ NCs (up to 1024 μg/mL) without NIR irradiation showed no significant bacterial inhibition. The study successfully proved the anti-infection performance of Au–Ag@SiO_2_ NCs in the rat model of wound infection, suggesting that photothermal MSN based nanomaterials can effectively clear the bacterial biofilm*.*

### Photodynamic therapy assisted with MSNs-based antibacterial

Photodynamic therapy (PDT) against bacteria is also regarded as a high-efficiency alternative approach to eradicate bacteria both in vitro and in vivo [106, 107]. PDT is a photochemical reaction that primarily relies on photosensitizers (PSs) under appropriate irradiation to achieve antimicrobial therapy through the production of cytotoxic ROS, including hydroxyl radicals, superoxide, or singlet oxygen (^1^O^2^) [108, 109]. The ROS generated by photosensitizers (PSs) can be targeted to the bacteria's internal and external structures, which cause irreversible oxidative damage to bacterial cell membranes and DNA molecules, leading to leakage of cell contents and enzyme inactivation [110]. Thus, PDT antibacterial therapy is an unspecific strategy that causes the bacterial death rather than develop drug resistance.

Chlorin e6 (Ce6) has been used frequently as a photosensitizer for PDT, which generates ^1^O_2_ when exposed to NIR light [111]. However, poor water solubility and negative charge make it difficult to contact bacteria and biofilm, which limits the antibacterial efficiency of PDT. To improve antibacterial performance, Yan et al. prepared an ultrathin chitosan-coated Ce6-loaded hollow mesoporous silica nanoparticles (UHSN@CS-Ce6) to enhance the loading capacity and photodynamic properties of Ce6 (Fig. 4E) [112]. The nanoparticles exhibited high loading efficiency, which significantly increased the ROS yield of Ce6 and effectively adhered to *S. aureus* biofilm, resulting removal rate of *S. aureus* biofilm up to 81% (Fig. 4F). Further, UHSN@CS-Ce6 accelerated skin wound healing in an *S. aureus* infection rabbit model. The phenothiazine photosensitizer methylene blue (MB) was positively charged at physiological pH which adheres to the bacterial cell wall by electrostatic interaction. Based on the properties of MB in the physiological environment, Oriol et al. designed a MSNs functionalized with amino or mannose to adsorb MB, effectively killing *E. coli* and *Pseudomonas aeruginosa* under red light irradiation and reducing MB toxicity [113].

Currently, photodynamic antimicrobial therapy (PDT) using lanthanum-doped upconversion nanoparticles (UCNP) as an energy donor in response to near-infrared light has various advantages, such as strong tissue penetration, broad antimicrobial spectrum, and low drug resistance, but was still limited by low efficacy. Li et al. proposed a novel bioinorganic nanohybrid that enables enzymatic-photodynamic effects on bacteria [114]. In this nanohybrid, UCNP encapsulated dendritic mesoporous silica was used as the carrier of photosensitizer MB and macromolecular lysozyme (LYZ) (Fig. 4G). The antibacterial mechanism of the hybrid nanoplatforms was first disrupted by LYZ, which exposes the bacteria to a large amount of ROS produced by MB to achieve the synergistic antibacterial effect of LYZ-PDT. The efficacy of the treatment on deep tissue MRSA infection was investigated, which LYZ or PDT treatment groups were effective in promoting healing. Surprisingly, the combination of LYZ and PDT was the most effective, without causing any side effects.

Rose Bengal (RB) is considered to be a promising PS due to the visible absorption band in the range of 480–550 nm and the high yield of single-line oxygen. Gehring et al. [115] successfully prepared photo-triggered silica nanoparticle systems by covalently combining RB/NO with thiol-functionalized PMO-type monodisperse silica [115]. In this nanosystem, nitric oxide (NO) was combined with superoxide radicals (O^2−^) and simultaneously released singlet oxygen to act as reactive oxygen species (ROS), which significantly enhances the antibacterial activity. But improving the production efficiency of single-line oxygen is the key to achieving photodynamic antibacterial activity. Protoporphyrin IX (PpIX) is a promising natural photosensitizer, but low solubility in physiological media prevents direct application to PDT. Thus, Zampini et al. prepared different porosity silica protoporphyrin IX (PpIX) nanocomposites and evaluated single-linear oxygen production as well as bacterial inactivation efficiency [116]. The studies showed that silica-protoporphyrin IX (PpIX) nanocomposites with larger pores have higher oxygen production in the single-linear state and greater antibacterial efficiency.

However, those PSs loaded MSNs materials easily separate from the infected sites, potentially leading to serious complications and inflammation [117]. To address this challenge, Sun et al. utilized electrospinning technology to create a self-enriched antibacterial membrane based on zein and polycaprolactone (PCL), MB loaded MSNs that has been fluoroalkane functionalized as the bactericidal ROS generator [118]. This antibacterial film can achieve PDT antibacterial adhesion synergy, revealing that both *S. aureus* and *E. coli* survived at low rates (≤ 3%) when exposed to visible light (660 nm, 20 min). Therefore, this MSN-based antibacterial composite membrane can be widely used in bacterially infected areas. Cinnamaldehyde (CA) is a natural antibacterial active ingredient, but poor water solubility and volatility significantly hinder the clinical application. Combining the excellent antibacterial properties of cinnamaldehyde (CA) with the copper sulfide nanoparticles (CuS NPs) of photothermal properties, a multifunctional nanoplatforms of silica nanospheres (SiO_2_ NSs) was constructed [119]. This mesoporous silica nanoparticles (SiO_2_@CA@CuS) first attach to the negatively charged bacterial surface, then rapidly kill the bacteria by the synergistic release of CA and heat generation when exposed to NIR light. This nanoplatform was important in the development of new, biocompatible, efficient, and synergistic antibacterial strategies.

### Photo-synergized therapy assisted with MSNs-based antibacterial

The single PDT therapy process kills bacteria mainly by producing massive amounts of ROS [120]. However, excessive amounts of reactive oxygen species could lead to normal cell inflammation and necrosis. Therefore, the integration of PDT and PTT can effectively reduce the side effects of single-modality antibacterial therapy and provide a way to develop a safer antibacterial strategy for phototherapy [121, 122]. The AuNRs@Cur nanoparticles were PTT/PDT bimodal antibacterial nanocomposites that used curcumin (cur) as the photosensitizer and gold nanorods encapsulated in silica as the photothermal carrier (Fig. 4H) [123]. The AuNRs@Cur has the strongest antibacterial effect on both *S. aureus* and *E. coli* when exposed to dual-light, which indicated that synergistic photodynamic-photothermal strategy can significantly increase bactericidal activity, and treat pathogenic diseases brought on by drug-resistant bacteria.

Similarly, researchers fabricated a Chlorin-e6 (Ce6) conjugated mesoporous silica nanoparticles (called AuNR@SiO_2_-NH_2_-Ce6) that exhibited synergistic photothermal and photodynamic effects [124]. This AuNR@SiO_2_-NH_2_-Ce6 could target bacterial, and subsequently release the Ce6 loaded in mesoporous silica by photothermal effect. This allows the generated ROS to penetrate the bacterial cell membrane or directly into the bacterial interior, thus effectively killing the bacteria. It not only disrupted the bacterial cell membrane integrity but also promoted the penetration and accumulation of external antibacterial agents in bacteria when exposed to light, which had a wide guideline for the design of MSN- based antibacterial nanoplatforms.

Besides the combined antimicrobial strategy of PTT and PDT, the effective therapeutic approaches such as chemodynamic therapy (CDT) with photothermal therapy (PTT) or photodynamic therapy (PDT), and gas therapy in combination with MSNs nanoplatforms have become a hot research topic in the treatment of antibacterial infections [125]. Due to the low bacterial resistance and high antimicrobial efficiency, it has been widely researched as an alternative therapy to antibiotics. Typically, the conversion of hydrogen peroxide (H_2_O_2_) into toxic hydroxyl radicals (·OH) using Fenton or Fenton-like reactions is used in chemodynamic therapy (CDT) to kill bacteria [126]. Besides, PTT converts light into heat, which causes protein denaturation by disrupting cell membranes, leading to bacterial death. However, for the treatment of deep bacterial infections at specific sites, such as periodontal disease, it is difficult to achieve efficient biofilm removal through the use of photo-based therapies. Therefore, the introduction of gas therapy using endogenous gas molecules such as carbon monoxide (CO), hydrogen sulfide (H_2_S), and nitric monoxide (NO) may facilitate the treatment of bacterial infections at deep tissues [127].

It was found that NO not only reacted with ROS to generate nitrite (ONOO^−^) radicals, which enhanced lipid peroxidation and disrupted bacterial membranes to enhance antimicrobial activity, but also altered extracellular matrix polysaccharides, which in turn induced physical rupture of bacterial biofilms. To this end, Qi et al. reported a novel synergistic antimicrobial strategy combining PDT, PTT, and gas therapy (Fig. 4I) [128]. This antibacterial and anti-inflammatory bifunctional nanoparticle was constructed by using the well-established core–shell structure of photothermal gold nanorods and mesoporous silica as a drug carrier, modified with indocyanine green (ICG), as well as the introduction of an NO donor. This triple function nitrogen oxide nanogenerator GNRs@mSiO_2_-SNO/ICG NPs can effectively remove periodontal biofilms and inhibit inflammation, as well as effectively kill periodontal bacteria by altering the permeability of cell membranes and preventing pathogenic biofilm formation.

## Physical interaction induced antibacterial therapy

**Fig. 5:**
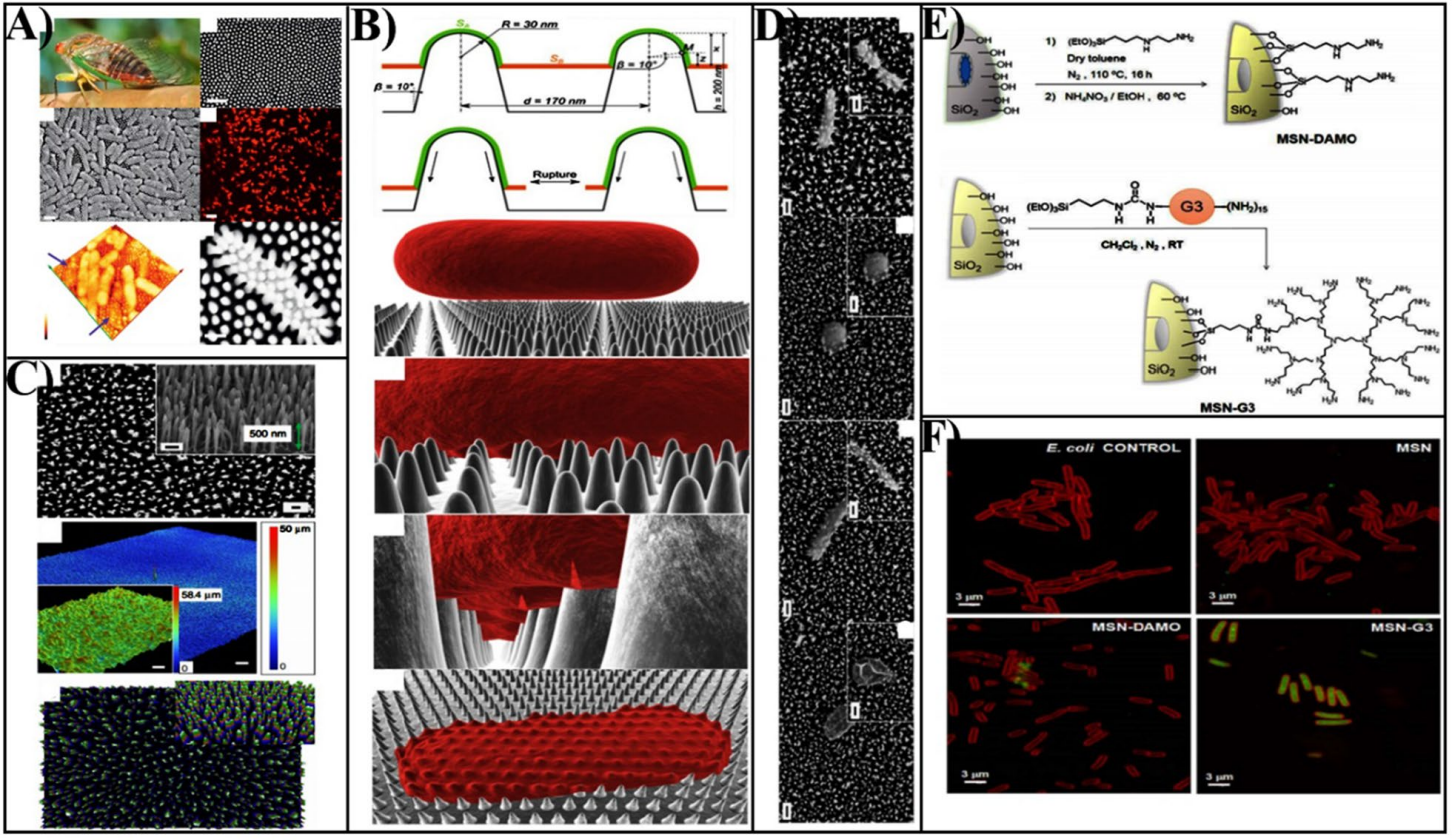
**A** A natural antibacterial idea inspired by the nanostructures present on cicada wings the surfaces. Reproduced with permission from Ref. [130] Copyright 2013, Elsevier Ltd. **B** The biophysical model of the interaction between nanopillars and bacterial cells on the wing surface of the cicada (P. claripennis). Reproduced with permission from Ref. [132] Copyright 2013, The Biophysical Society. **C** Characterization of black silicon. **D** It was observed that B. subtilis vegetative cells and spores of B. subtilis were significantly disrupted by interaction with bSi. Scale bars, 200 nm. Reproduced with permission from Ref. [133] Copyright 2013, Macmillan Publishers Limited. **E** Schematic diagram of MSN-DAMO and MSN-G3 nanomaterials. **F** The internalization of MSN, MSN-DAMO, and MSN-G3 materials towards Gram-negative *E. coli*. Reproduced with permission from Ref. [135] Copyright 2018, Elsevier Ltd

Besides the chemical damage of cell membrane through direct contact between MSNs and bacteria, the physical damage also is an important mechanism for MSNs based nanomaterials. Due to the unique physiochemical characteristics of nanomaterials, including surface charge and topology, the direct contact of nanomaterials with bacteria can also damage bacterial cell membranes (“contact killing”) [129]. The highly ordered arrays of nanopillars of cicada wings surface are representative of the first example of contact-killing nanomaterials, which are capable of killing bacteria based on contact with their physical surface structure alone (Fig. 5A) [130]. Due to the adhesion of bacteria onto the nanopillar structures on the wing's surfaces, the bacterial cell membrane suspended above the nanopillars was sufficiently stretched and mechanically ruptured depending on the physic-mechanical effect of the nanopillars instead of surface chemistry (Fig. 5B) [131, 132]. As inspired by the cicada wing’s antibacterial surface, Ivanova et al. designed biomimetic silicon-based nanomaterials that contain high aspect ratio nano protrusions-linked structures on its surface for generating a mechanical bactericidal effect, the physical disruption to the bacterial cell membrane (Fig. 4C, D) [133]. Therefore, the antibacterial activity of MSNs can be obviously enhanced dependent on the surface topographic properties.

Moreover, the surface positive charged MSNs could also enhance the interaction with the bacterial surface, since bacteria typically have negatively charged cell membranes composed of lipid layers and peptidoglycan [87]. It was known that the cationic surfactant or polymers micelles have been proven to enhance antibacterial properties by increasing electrostatic interactions with the bacteria wall or biofilm with negatively charge [134]. González et al. fabricated a new class of antibacterial agent where MSNs were decorated with polycationic polypropylenemine dendrimers (G3) loaded with LEVO [135]. Compared to [3-(2-aminoethylamino) propyl] trimethoxy silane 95% (DAMO) modified MSN, G3 grafted MSNs can penetrate bacteria walls, which allows effective internalization into Gram negative bacteria (Fig. 5E, F). The powerful electrostatic forces generated when the MSNs adhere with bacteria could seriously damage bacterial cell membranes and result in subsequent bacterial death. Notably, extremely low positive charge densities are invalid in contact-killing, while extremely high charge densities can also damage mammalian cell membranes.

## Targeting-directed MSNs-based antibacterial therapy

The majority of antibacterial agents used to treat bacterial infectious diseases lack targeting, which leads to harmful side effects and low therapeutic efficacy since they can hardly accumulate at the desired infection site after administration [136, 137]. As a novel antibacterial nanocarrier, MSNs can be easily chemically modified to target bacteria for further treatment. Therefore, delivery efficiency could be improved while the dose and frequency of administration could be decreased by targeted MSNs delivery systems.

### Engineering MSNs to target planktonic bacteria

The bacterial cell wall is a protective layer consisting primarily of peptidoglycans and glycolipids, these unique components can be used as excellent targets of MSNs for bacteria [138, 139]. Thus, by selecting the right targeting molecule, the targeted MSNs can be guided into the interior of a specific type of bacteria. Based on the above goals, various research has focused on using various targeted materials to decorate the outer layer of MSNs to particularly recognize bacteria and effectively adhere to bacterial surfaces (Table 2).

**Table 2:** Overview of bacterial-targeted MSNs delivery systems

Type of targeting	Targeting ligand	Bacterial	Type of device	Refs.
Electrostatic interaction	Quaternary ammonium polyethyleneimine (QPEI)	*P. syringaepv. Lachrymans, Clavibacter michiganensis subsp. michiganensis*	Ag@MSN-QPEI	[140]
Bionic recognition	Outer membrane vesicles (OMVs)	*S. aureus、E. coli*	Rif@MSN@OMV	[142]
Aptamers recognition	Trehalose	*Mycobacteria*	HOMSNs-Tre-INH	[143]
	SA20hp	*S. aureus*	Aptamer-gate NP	[144]
	Lactobacillus monocytogenes aptamer	*L. monocytogenes*	AptBACNPs	[145]
Antigen–antibody recognition	Anti-*S. aureus* antibody	*S. aureus*	Ab@S-HA@MMSNs	[146]
	*Staphylococcus aureus* (*S. aureus*)-specific aptamer	*S. aureus*	RAHMSN@AGNR	[147]
Peptides recognition	Ovotransferrin-derived antibacterial peptide (OVTp12)	*E. coli*	MSNs@OVTp12@Gen	[93]
	Peptide UBI29-41	*S. aureus*	MSN@D&U@V	[150]
Carbohydrates recognition	Gluconamide moieties	*E. coli*	glc-SiO_2_NPs	[151]
	Trehalose	*M. smegmatis*	M-PFPA-Tre-INH	[152]

#### Electrostatic interaction-based targeting

The MSN with positively charged can adhere to the outer membrane of bacterial walls with negative charge through electrostatic interactions, interfering with bacterial metabolic processes as well as causing perforation or even membrane leakage. Furthermore, it has been demonstrated that the MSN surface with positively charged enabling it easier to internalize for bacteria. Silver nanoparticles (AgNPs) exhibit significant broad-spectrum antibacterial activity.

**Fig. 6:**
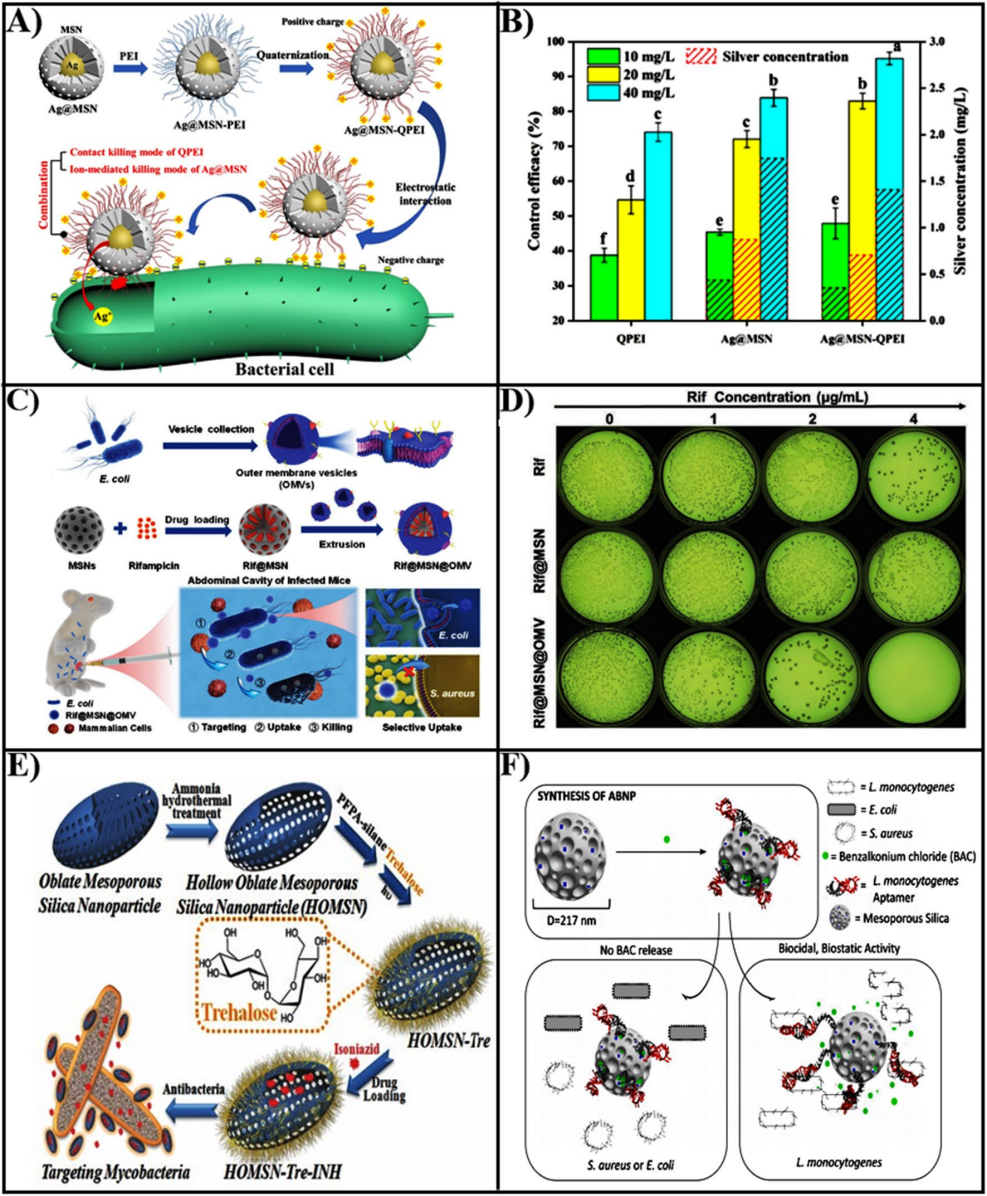
**A** The preparation process of Ag@MSN-QPEI nanoparticles and the mechanism of electrostatic adhesion to bacteria. **B** The antibacterial effects of QPEI, Ag@MSN and Ag@MSN-QPEI at 10, 20 and 40 mg/L concentrations against *P. syringae pv. lachrymans*. Reproduced with permission from Ref. [140] Copyright 2021, American Chemical Society. **C** Illustration of the design of a bionic nano-delivery system for the treatment of bacterial infections. **D** The inhibition of *E. coli* by Rif, Rif@MSN, and Rif@OMV@MSN nanoparticles. Reproduced with permission from Ref. [142] Copyright 2021, Wiley–VCH GmbH. **E** The construction and targeting of HOMSN-Tre-INH nanosystems to bacteria. Reproduced with permission from Ref. [143] Copyright 2015, WILEY–VCH. **F** Illustration of the specific inhibition of *L. monocytogenes* by BAC loaded mesoporous silica nanoparticles. Reproduced with permission from Ref. [145] Copyright 2021, Elsevier B.V

However, the MSN surface with negative potential has electrostatic repulsion with bacterial cell membranes, which limits antibacterial activity. To address the limitation, Niu et al. utilized cationic antibacterial polymer quaternary ammonium salt (QPEI) modified MSN loaded with AgNPs. [140]. These nanomaterials with a strong positive surface charge, which exhibited a favorable bacterial targeting effect and could adhere to the surface of negatively charged bacterial biofilm to release silver ions (Fig. 6A). The nanoparticles (Ag@MSN-QPEI) loaded with AgNPs prepared by QPEI modified MSN exhibited superior antibacterial activity and long effective action time at the same silver ion concentration (Fig. 6B).

The polycationic dendrimers with positively charged amine groups could effectively bind bacterial cell walls, enhancing membrane permeability of bacterial and internalization within the bacteria [141]. González et al. constructed a "nano-antibiotic" system by using third generation (G3) poly (acrylimide) dendrimers grafted onto an MSN surface loaded with levofloxacin (LEVO) [135]. The positive charge carried by the polycationic dendrimer acted as an internalizing agent, which allows electrostatic interaction with the bacterial cell wall, penetration of the cell wall, and internalization into the bacteria. Therefore, this MSN based nonantibiotic delivery system possessed high permeability to Gram-negative bacterial membranes. And the disruption of bacterial cell walls by G3 dendritic macromolecules and the bactericidal action of LEVO could generate synergistically antibacterial effects against Gram-negative bacterial biofilms.

#### Bionic recognition-based targeting

Based on inspiration from nature, the use of natural cell membranes as coatings for MSN based nanomaterials, can enhance targeting and antibacterial activity. The outer membrane vesicles (OMVs) of *E. coli* primarily composed of lipopolysaccharides, peptidoglycans, membrane proteins, and nucleic acids, are similar in composition to bacterial membranes. Hence, OMVs can act as bacterial targeted agents, enhanced fuse with bacterial cell membranes, and bacterial uptake of loaded antibiotic nanoparticles. Wu et al. constructed a biomimetic nanoplatform (Rif@MSN@OMV) utilizing outer membrane vesicles (OMVs) isolated from *E. coli* as the shell and an MSN loaded with rifampicin (Rif@MSN) as the core (Fig. 6C) [142]. In vitro, the antibacterial activity when Rifampicin concentration of 4 µg/mL of Rif@MSN@OMV exhibited 99.8% inhibition of *Escherichia coli* within 24 h (Fig. 6D). Furthermore, a single injection of Rif@MSN@OMV enhanced infected mice’s survival and decreased the bacterial burden of intraabdominal fluid and organs in a mouse model of peritonitis. But, this bionic nano-delivery system had poor sensitivity to other bacteria, which only responded against *E. coli.* To treat multidrug-resistant (MDR) bacterial infections, this approach could expand the application OMVs encapsulated MSN delivery systems.

#### Aptamers recognition-based targeting

Aptamers are functional oligonucleotides that have a strong affinity for a variety of targets, including proteins, peptides, carbohydrates, small molecules, toxins, and even living cells, so frequently utilized as precise targeting agents for drug delivery systems. Trehalose, a glycolipid found in the cell wall of *mycobacteria*, can be employed as a targeted ligand for the microorganism. Thus, Hao et al. presented evidence demonstrating the selective targeting and eradication of *mycobacteria* by isoniazid (INH)-loaded hollow oblate mesoporous silica nanoparticle (HOMSNs-Tre-INH) modified with alglucose (Fig. 6E) [143]. The targeted adhesion bacterial results of HOMSNs-Tre-INH showed that no particles were seen on either *E. coli* or *Staphylococcus epidermidis*, while nanoparticles were only seen on the surface of *mycobacteria*, further proving that alglucose was a targeted agent specifically for *Mycobacterium*.

The SA20hp aptamer had a great affinity for the surface antigens of *S. aureus* and performed targeted to bacteria. Kavruk et al. presented a targeted nanoplatform based on SA20hp aptamer modified MSN loaded with vancomycin (VAN), capable of selectively targeting and eradicating *Staphylococcus aureus* [144]. The efficacy of antibiotics against *Staphylococcus aureus* had more than 15-fold increased, which was attributed to this nanoplatform could selectively identify the bacteria and release antibiotics via an antigen-triggering mechanism. The aptamer molecular gate structure could provide specific targeting for benzalkonium chloride (BAC), Sudagidan et al. used *Lactobacillus monocytogenes* aptamers as targeting and capping agents for MSN loaded with benzalkonium chloride (BAC) (Fig. 6F) [145]. The BAC-modified aptamer functionalized nanoparticles (AptBACNP) displayed specific antibacterial activity only against *Lactobacillus monocytogenes*, which were ineffective on *S. aureus* and *E. coli*. The antibacterial efficiency of AptBACNPs was 4 and 8 times greater against moderately resistant and sensitive strains than free BAC, demonstrating AptBACNPs could specifically transport BAC molecules to the inside of bacteria.

#### Antigen–antibody recognition-based targeting

The antibodies are extremely specific ligands that can bind to antigens on the surfaces of bacteria with a high affinity. Utilizing this outstanding performance, Xu et al. prepared loaded vancomycin (VAN) magnetic mesoporous silica nanoparticles (Ab@S@HA@MMSNs) modified with anti-*S. aureus* antibody [146]. Then, it was fixed into a magnetic glassy carbon electrode (MGCE) to achieve sensitive, quick, precise testing, and elimination of bacteria in the blood. When the increased amount of *S. aureus* arrives at MGCE, the antigen–antibody specifically binds between the *S. aureu*s in the solution. The anti-*S. aureus* antibody of the MGCE surface could cause a change of the electrochemical signal, thus accurate determination of the *S. aureus* content in the solution. Moreover, the hyaluronidase secreted by *S. aureus* hydrolyzes the antibodies on the mesoporous silica capping layer, which releases antibiotics to kill the bacteria. The platform was a highly selective immunosensor for *S. aureus*, with no significant change in current value when detected *E. coli* and *Pseudomonas aeruginosa*, while the current value for *S. aureus* changes significantly with concentration. The platform provides a perfect electrochemical response against *S. aureus* in the range of 10–10^10^ CFU/mL, which has a limit of detection for three colony forming units (CFU) per mL, and has sensitivity, outstanding selectivity, and reproducibility. The integrated platform enabled the effective eradication of *S. aureus*, which enables accurate diagnosis and effective treatment of *S. aureus* infections disease.

**Fig. 7:**
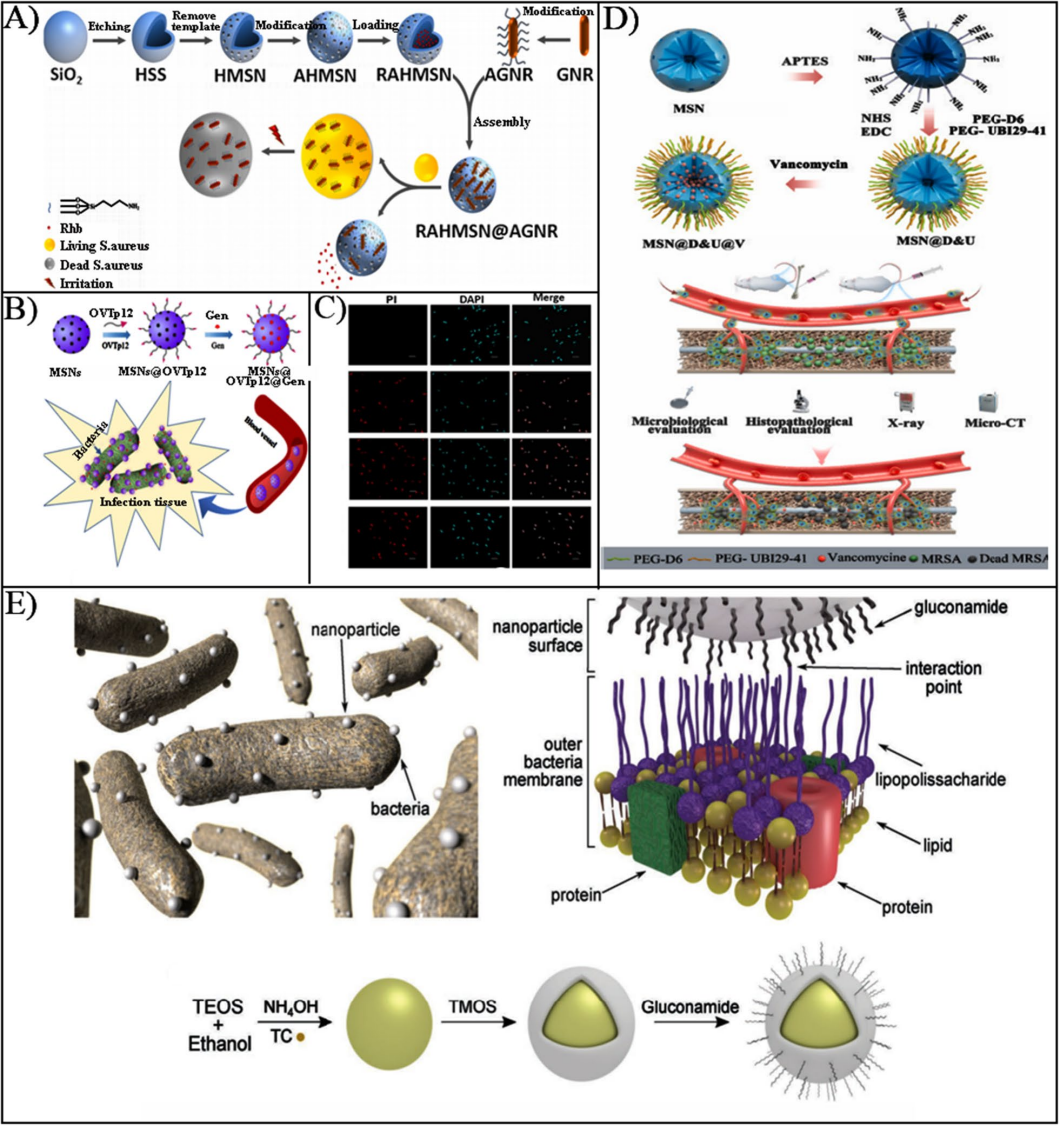
**A** The construction process of RAHMSN@AGNR nanoparticles based on gold nanorods (AGNR) modified with specific aptamers. Reproduced with permission from Ref. [147] Copyright 2021, Elsevier Ltd. **B** Schematic representation of the synthesis of MSNs@OVTp12@Gen nanoparticles and their targeting to bacteria. **C** The inhibition of *E. coli* by Gen, MSNs@Gen, and MSNs@OVTp12@Gen nanoparticles. Reproduced with permission from Ref. [93] Copyright 2021, American Chemical Society. **D** Illustration of the synthesis of MSNs with dual targeting and their biological function research. Reproduced with permission from Ref. [150] Copyright 2022, Elsevier B.V. **E** Illustration of efficient design of carbohydrate-based mesoporous silica nanoparticles targeting Gram-negative bacteria. Reproduced with permission from Ref. [151] Copyright 2019, WILEY–VCH

Due to improving the gating specificity, aptamers are generally utilized act as gatekeeping on–off, a bifunctional smart nanoplatform for bacterial detection and sterilization electrostatically was designed. The surface of aminated hollow mesoporous silica loaded with rhodamine B was modified with a gold nanorod (AGNR) of *Staphylococcus aureus*-specific aptamer (GNR) (Fig. 7A) [147]. When *S. aureus* was present, the aptamer preferentially targeted the bacteria, which caused AGNR to shed and rhodamine B to release for *S. aureus * fluorescence detection. The sensitivity was excellent when the concentration of *S. aureus* was in the range of 6.5 × 10^2^ CFU/mL ~ 6.5 × 10^7^ CFU/mL. When exposed to NIR, AGNR exerted a photothermal bactericidal effect, specifically blinded and killed *Staphylococcus aureus*, and the sterilization efficiency could reach 100% with 2 W IR light irradiation for 5 min.

#### Peptides recognition-based targeting

Peptides, which are combinations of small molecules and proteins, are a great alternative for active target modification ligands for nanoparticles due to high targeting, excellent biocompatibility, high safety, facile chemical modification, and wide source. The new peptide (OVTp12) was high sensitivity and specificity for bacterial cell membranes, Ma et al. devised gentamicin-loaded mesoporous silica nanoparticles (MSNs@OVTp12@Gen) that modified a new peptide (OVTp12) extracted from egg white oval transferrin for targeted delivery to bacteria (Fig. 7B, C) [93]. These nanomaterials could be used to treat internal bacterial infections by targeting *E. coli* to achieve a killing effect.

Due to the inability of conventional antibiotic therapy to specifically target the location of bone infection, bacterial-associated infections brought on by bone implants were more likely to result in limb sequelae and mortality. The synthetic peptide D6 was regarded as a potential target of the bone infections therapeutic molecule [148, 149]. Furthermore, Peptide UBI_29-41_ was a commonly bacterial targeting peptide with six positively charged residues that targeted *Staphylococcus aureus* with a strong negative charge. Therefore, to target and control vancomycin release at the bone infection site, Nie et al. designed a bone and bacterial dual targeting nanoparticle (MSN@D&U@V) by combining D6 and UBI29-41 peptides (Fig. 7D) [150]. In the rat model, MRSA-induced orthopedic implant-associated infection, the antibacterial activity of MSN@D&U@V dramatically suppressed the growth of femoral bacteria and successfully decreased femoral bone damage.

#### Carbohydrates recognition-based targeting

As the main source of carbon for bacteria, carbohydrates are involved in the metabolic and transport processes of bacteria, which are widely existing on the surface of bacterial cells. Therefore, using carbohydrates as targeting agents and exploiting carbohydrate-mediated metabolic mechanisms, designing nanoparticles targeting bacteria is effective. In pursuit of new strategies to combat bacterial resistance, Capeletti et al. designed MSNs encapsulated by glucosamine to target lipopolysaccharides in the outer membrane of *E. coli*, enabling the drug could effective delivery to bacterial cell wall (Fig. 7E) [151]. There exhibits excellent stability of glucosamine-functionalized MSNs and low toxicity to mouse embryonic fibroblasts (NIH3T3), which made it suitable for targeted *E. coli*, and preventing particle aggregation and the adsorption of non-specific protein.

The multidrug resistant *Mycobacterium* tuberculosis poses a serious threat to human health. Isoniazid (INH), a first-line treatment for tuberculosis, prevents *Mycobacterium avium* from synthesizing cell walls, but severe toxicity prevents it from being used in clinics. Thus, the search for a novel strategy for rapidly and specifically the delivery of drugs to achieve higher local concentrations and minimize side effects is essential and urgent. Zhou et al. designed MSNs loaded with INH encapsulated by functionalized alginose for selective target killing of drug-resistant *Mycobacterium avium* [152]. The results of selectivity and antibacterial activity against *Mycobacterium avium* demonstrated that INH concentration of 3–4 mg/mL MSNs functionalized with alginate had complete growth inhibition. In contrast, only INH concentrations of 4.5–5 mg/mL of alginate-free MSNs functionalization are effective. These results indicated that trehalose conjugated at the surface of MSNs played an important targeting role on the bacteria.

### Engineering MSNs to target biofilm matrix

Bacterial biofilms are intricate bacterial colonies encased in a protective extracellular polysaccharide (EPS) matrix that resist the action of antibacterial medications and drastically decrease the effectiveness of antibiotics as compared to planktonic cells (up to 100-fold) [153]. Once established, the bacterial biofilm served as a depot for bacteria, boosting susceptibility to antibacterial agents and their capacity to elude the host immune system, leading to the development of persistent and recurrent infections. A variety of targeted MSNs can effectively eliminate planktonic bacteria. However, when bacteria form biofilms, the situation becomes much more challenging. Whereas it is not impossible to affect both EPS and bacteria inside of the biofilm, as EPS is a key component of the biofilm matrix. Therefore, MSNs-based targeted nanoplatform for bacterial biofilms, which can disrupt EPS, penetrating the bacterial biofilms, and then releasing antibacterial agents to kill bacteria within the bacterial biofilm, is a promising approach to eradicating bacterial biofilms (Table 3).

**Table 3:** Overview of MSNs-based delivery systems to targeted elimination of bacterial biofilm

Strategies	Targeting ligands	Bacterial biofilm	Nancarriers	Refs.
Ligand-targeted	Lectin concanavalin A (ConA)	*E. coli*	MSNConA@LEVO	[155]
	Lysostaphin、serrapeptase、DNase I	Methicillin-resistant *S. aureus* (MRSA)	Lys/Ser/DN@MSN	[158]
Electrostatic interactions	Polyethyleneimine (PEI)	*S. aureus*	AHMSN@GA@PEI@Cur	[160]
	*N*-(2-aminoethyl)-3- aminopropyltrimethoxysilane (DAMO)	*E. coli*	MSN-DAMO@LEVO	[135]

#### Ligands target extracellular polymeric substances (EPS)

**Fig. 8:**
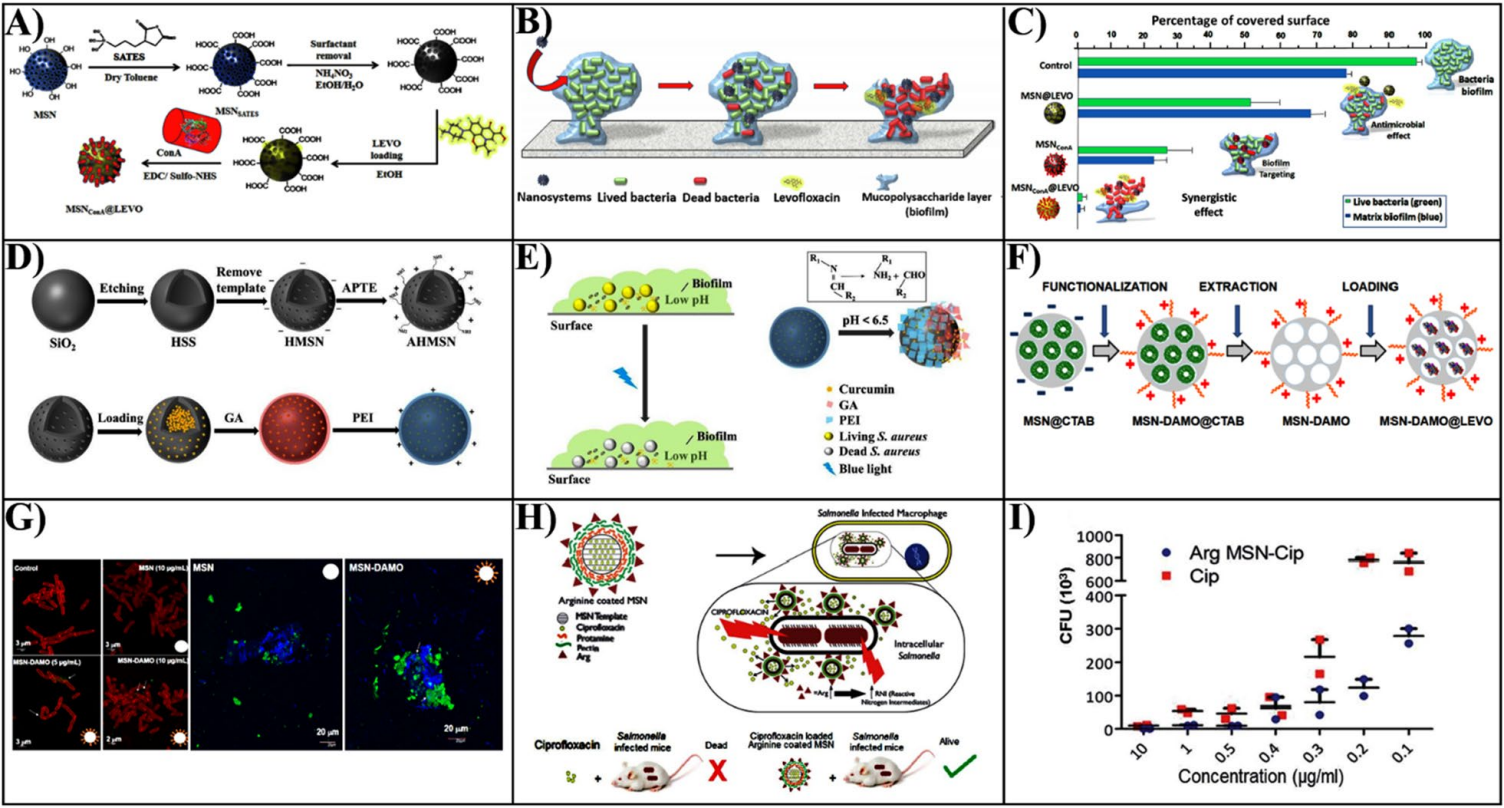
**A** The design of MSNConA@LEVO nanoparticles and evaluation for targeting bacterial biofilms. **B** MSNConA@LEVO nanoparticle targeted removal of biofilm. **C** Antibacterial activity of MSNConA@LEVO nanoparticles against Gram-negative *Escherichia coli* biofilms. Reproduced with permission from Ref. [155] Copyright 2021, Elsevier Ltd. **D** Illustration of design of the AHMSN@GA@PEI@Cur delivery system. **E** Schematic diagram of pH-responsive killing of bacteria within biofilms by AHMSN@GA@PEI@Cur nanoparticles. Reproduced with permission from Ref. [160] Copyright 2022, MDPI, Basel, Switzerland. **F** Schematic design of MSN-DAMO@LEVO nanoparticles. **G** Imaging of nanoparticle-targeted adhesion to *Escherichia coli* cell walls and biofilms. Reproduced with permission from Ref. [45] Copyright 2018, De Gruyter Open. **H** Schematic diagram of Cip-Arg-MSNs nanoparticles targeting the treatment of bacterial infections within macrophages. **I** The bacterial growth inhibition of *Salmonella* by Cip and Arg-MSN-Cip. Reproduced with permission from Ref. [165] Copyright 2017, The Royal Society of Chemistry

The dense structure of EPS provides a physiological barrier to antibacterial drugs, which can be trapped by EPS, reducing the bacterial killing effect of antibacterial drugs. Therefore, acting on EPS will be an effective strategy to remove the bacterial film. For EPS, which mostly consists of proteins, polysaccharides, lipids, and eDNA, several previous studies have concentrated on using MSN to transport antibiotics to bacterial biofilms, lowering the cohesion and biofilm biomass of EPS, based on each attribute [154]. The lectin concanavalin A (ConA) is a glycoprotein that exist in a variety of organisms, which can recognize and bind to the glycan in the EPS of biofilm. In this case, Martínez-Carmona et al. constructed a novel targeted nanoplatform (MSNConA@LEVO) by covalently attaching ConA to the surface of MSN loaded with levofloxacin (LEVO) as a targeting aptamer via a carboxylic acid group (Fig. 8A) [155]. The targeting evaluation of the nanocarrier on the *E. coli* biofilm found that the internalization of MSNConA was dose-dependent, indicating the more ConA was present on the carrier of the outer surface, the more the targeting penetrated the inner biofilm matrix (Fig. 8B, C). In addition, the combination of ConA and levofloxacin (LEVO) generated a synergistic effect on biofilm eradication due to the ability of ConA to drive the targeted penetration of MSNConA@LEVO into the bacterial biofilm to release LEVO.

The degradation of the biofilm matrix by the proteinase K, plasmin, enzymes DNase I, amylase, serrapeptase, and dispersin B has been demonstrated in several bacteria [156, 157]. Therefore, a similar strategy for biofilm removal is to anchor lysozyme, serrapeptase, and DNase I to the surface of MSN, respectively [158]. The use of enzyme-functionalized amino MSNs almost completely removed the biofilms of MRSA and methicillin-sensitive *S. aureus* (MSSA), as well as prevented EPS diffusion. Therefore, there is a promising approach to improving the therapeutic potential of *S. aureus* biofilm-associated infections using enzyme-functionalized nanoparticles.

#### Electrostatic interactions target extracellular polymeric substances (EPS)

Based on the fact that EPS often has negative charges, a different approach is adjusting the electrostatic interactions between nanoparticles and biofilms [159]. In this line, Prof. Zhao group reported an amino-modified hollow mesoporous silica nanoparticle (AHMSN) that was loaded with the natural photosensitizer curcumin (Cur) (Fig. 8D, E) [160]. Then, the porous structure of the AHMSN has been sealed through a Schiff base reaction by glutaraldehyde (GA) and polyethyleneimine (PEI), which generated positively charged AHMSN@GA@PEI@Cur. The dependence on electrostatic adhesion between the positively charged nanoparticles and the biofilm leads to the release of Cur into the biofilm. The clearance rate of *S. aureus* biofilm was 98.20% when exposed to 450 nm blue light. The positively charged hollow mesoporous silica with strong permeability to bacterial biofilms, which light triggered the PDT effect played a synergistic antibacterial and antibacterial biofilm.

Other research groups have also proposed various similar biofilm targeting strategies, such as Prof. Vallet-Regí's team which reported various "nano-antibiotic" delivery systems (Fig. 8F, G) [45, 135]. Which mainly consists of positively charged MSN loaded with antibiotics that act as a targeting and permeation agent to bacterial biofilms. Therefore, the most common strategy is the modification of MSN using amination reagents to provide a positive charge and loading of levofloxacin (LVX), which enhances the capacity of the nanomaterials for targeting and penetrating *S. aureus* biofilms. In addition, modification of LVX-loaded MSN using cationic dendritic macromolecules as bacterial permeation targeting agents also showed high synergistic elimination of *E. coli* biofilms.

### Engineering MSNs to target bacteria-infected macrophage

Macrophages are a type of white blood cells that acts as the first line of defense for the host's innate immune system. Thus, the macrophages play an important role in recognizing and eliminating bacteria since infected tissues can seek assistance via the release of chemicals that attract macrophage [161]. But, the cytoplasm of the host macrophages can be used by a large number of pathogenic bacteria to replicate and evade the host's innate immune response. Due to the antibiotic’s poor ability to enter cells, the bactericidal concentration needed to kill intracellular bacteria is higher than to kill extracellular bacteria [162, 163]. Therefore, the strategy for the treatment of intracellular infections is to effectively deliver the antibacterials to the bacterially infected macrophages.

*Salmonella* readily invades the immune cells and survives in the internal environment, such as macrophages. Arginine is a critical regulator for the stimulation of macrophage responses to cellular immunity, facilitating macrophage phagocytosis and clearing intracellular pathogens through the generation of nitric oxide (NO) [164]. Considering infection of macrophages by *Salmonella* causes leads to increased uptake of arginine, Mudakavi et al. designed mesoporous silica nanoparticles (Cip-Arg-MSNs) with encapsulated *L-arginine* layer-by-layer on the surface of MSNs loaded with ciprofloxacin (CIP) (Fig. 8H) [165]. The Cip-Arg-MSN could be facile internalized by macrophages as well as a low toxicity, and compared to free ciprofloxacin, which increases antibacterial activity by 200%. (Fig. 8I). Vancomycin was known to be an effective antibiotic against bacteria due to the skeleton that could through hydrogen bonding interaction bind to the terminal D-alanyl-D-alanine portion of Gram-positive bacteria. The vancomycin (VAN) grafted onto the mesoporous silica nanoparticles (MSNs ⊂ VAN) surface can be effectively targeted to kill bacteria in macrophages [166]. And the in vivo evaluation of antibacterial activity in mice revealed a tenfold reduction within 5 days in the bacteria infected mice in the MSNs ⊂ VAN group, and the lowest inhibitory concentration of MSNs ⊂ VAN against *S. aureus* was 200 g/mL. Thus, it seems to be a promising strategy for the targeted delivery of antibacterial agents to treat bacterially intracellular infections.

## Challenges on translational application of MSNs-based antibacterial therapies

**Fig. 9:**
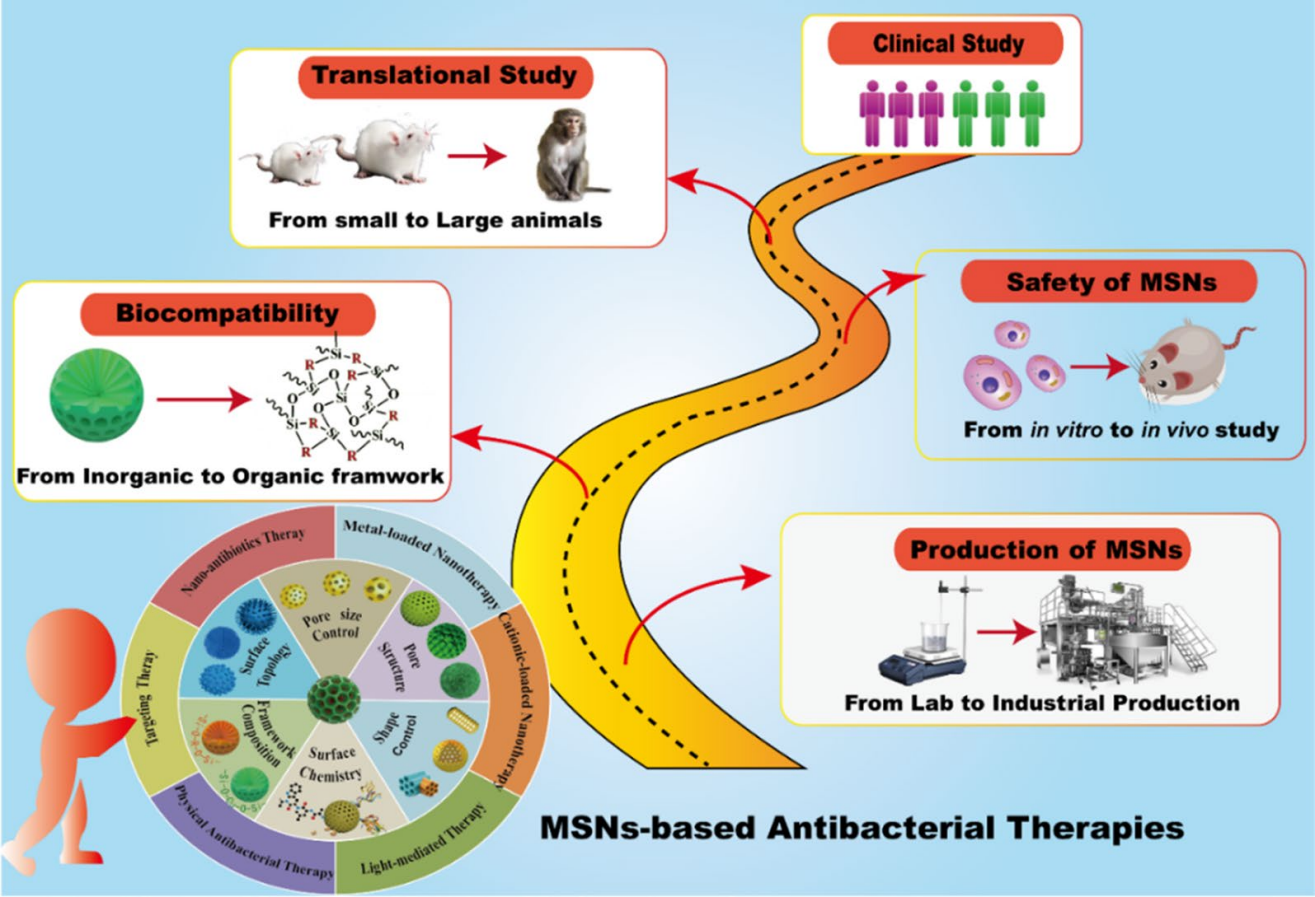
Schematic illustration on translational challenges of MSNs-based antibacterial therapies

MSNs-based nanotherapies present many merits of large surface area and porosity, adjustable surface charge and size of the particle, excellent stability, facilitation of functionalization, outstanding biocompatibility, and eradicating biofilms, which attract widespread attention in recent years for antibacterial applications. However, as displayed in Fig. 9, there is still a long way to go to translate these exciting scientific findings into clinical trials acquiring FDA approvals. To reach this aim, the safety and biocompatibility of the MSNs-based antibacterial nanomaterials are the main issues to be considered besides the efficacy. Firstly, the electrostatic interaction between the cell membrane and the silanol group (Si–OH) on the surface of the MSNs is one of the primary causes of cytotoxicity. This leads to membrane rupture and cell lysis as well as the generation of toxic ROS, which could lead to cell membranes lipid peroxidation, cell necrosis, or apoptosis [167, 168]. Furthermore, each molecule or material modified MSNs has different toxicity to cells and bacteria. For example, amine-modified MSNs is more cytotoxic than unmodified [169]. To be noted, with extensive research investigating the rate of degradation, excretion and propensity of accumulation of MSNs at in vivo level, the primary factors behind majority of biosafety issues remain less understood.

Designing organic–inorganic hybrid MSNs with appropriate physicochemical properties is one effective way to improve functionalities, particularly for a variety of complex multi-stimulus reactive antibacterial MSNs, by varying the surface charge, size, chemistry, and shape geometry of the particles. As a result, it is critically necessary to conduct extensive research on the biosafety and toxicology of MSNs-based antibacterial nanoplatforms, to provide a solid basis for clinical trial translation.

Furthermore, the difficulty of mass production of MSNs and poor batch-to-batch reproducibility stand for another challenge that hinder the translation and commercialization. At present, the synthesis of monodispersed MSNs can be easily achieved and controlled in the laboratory, but experimental parameters vary significantly when scaled up for industrial production. Especially, for the smart responsive targeted MSNs, the synthesis process is very complicated, poor reproducibility and high cost greatly limit industrial production. Therefore, the present design regarding intelligent mesoporous silica antibacterial nanoparticles should be simplified as much as possible. It is necessary to ensure the required efficacy and safety to establish a simple and effective MSNs-based antibacterial nanoplatform.

Remarkably, current preclinical studies on MSNs based nanomaterials have been mainly focused on small animals, with few experiments in dogs, pigs, or other large animals. However, there are significant differences in the physiological environment between small animals and humans. Therefore, experimental data on the pharmacokinetics, biodistribution, accumulation, and metabolism of MSNs in vivo obtained acquired only from small animal models do not provide an accurate and reliable basis for clinical translation. It is crucial to obtain further data on the biological properties of MSNs nanomaterials to guide subsequent clinical trials. However, obtaining large laboratory animals and the high cost poses a challenge to translation research.

Moreover, limited attention has been paid on the setting of standard evaluation criteria and methodologies for antibacterial nanotherapies, leading to difficulties in assessing the performance of the therapies and applying them to a broader extend. Therefore, regulations and policies from relevant institutions are critical to accelerate the clinical translation of MSNs based nanotherapies.

## Conclusion

The antibiotic resistance and the bacteria biofilm formation are the main causes of hard-to-cure of bacterial infections. MSNs-based nanomaterials have emerged as an effective approach to overcome this problem by improving the effectiveness of existing antibiotics or generating entirely novel antibacterial strategies. Owing to the good biocompatibility and easily adjustable physicochemical structures, MSNs have shown great potential in treating bacterial infections. Antibacterial agents (antibiotics, metal actives, polymer, and peptides etc.) can be functionalized or loaded in MSNs, which can significantly enhance the antibacterial performances compared to these agents alone. To synergize the antibacterial performance, photo-therapies including photodynamic and photothermal therapy already combined with the MSNs-based nanotherapy, allowing efficient killing of bacteria via varied mechanisms. Targeted antibacterial therapy also stands out due to the simultaneously enhanced bactericidal efficiency at infection site and reduced side effects.

Due to the high complexity of bacteria-induced infection diseases, it remains to be very challenging to promptly control bacterial infection through any single therapeutic approach, such as the direct administration of antibiotics. Targeting delivery and controlled release of antimicrobial agents would be an ideal strategy to enhance current therapies, while to reduce resistance issue, combined therapies have been a widely adopted route. To synergize the antibacterial performance, photo-therapies including photodynamic, photothermal therapy already and gas therapy can be combined with the MSNs-based nanotherapy, allowing efficient killing of bacteria via varied mechanisms. Therefore, a series of multifunctional MSNs antimicrobial nanoplatforms with the combination of targeted delivery, chemotherapy, photothermotherapy, and gas therapy have become a hotspot for development by introducing novel functional nanoparticles and their functionalization.

Compared to conventional nanocarriers, the versatile design of multifunctional MSNs nanoplatforms can be very promising in terms of on-demand drug release, enhanced drug accumulation at the site of infection, and improved interaction with bacterial cells. Although MSNs-based nanomaterials have been extensively researched, major focus has been drawn in the delivery function rather than turning them into bioactive materials to enhance the therapeutic performance. Therefore, the design and construction of multifunctional MSNs nanoplatforms integrating diagnostic and antimicrobial therapeutics have great potential for future development. As a major challenge of many antimicrobial agents, most of them can only be potent for a particular type or types of bacteria, owing to the significant differences in pathogen properties and their killing mechanisms. How to design MSNs-based multifunctional nanoplatforms that could accommodate multiple antimicrobial agents with several bacterial killing paths would be greatly favored for the next generation of antibacterial agents with enhanced versatility against bacteria or biofilms. This review comprehensively summarized and analyzed the design strategy of MSNs-based nanotherapies for bacteria and biofilm eradication, which aims to inspire the further development of novel MSNs-based nanoplatforms for infection control.

## Data Availability

Not applicable.
